# Antiviral and Anticancer Activities of Stingless Bee Propolis from *Tetragonula drescheri* and *Tetragonula pagdeni*: Toward Development of Prototype Healthcare Pharmaceuticals

**DOI:** 10.3390/ijms27093855

**Published:** 2026-04-27

**Authors:** Karnjanee Greegrainuch, Wiratchanee Kansandee, Chamsai Pientong, Tipaya Ekalaksananan, Jureeporn Chuerduangphui

**Affiliations:** 1 Department of Microbiology, Faculty of Science, Kasetsart University, Bangkok 10900, Thailand; karnjanee.g@ku.th; 2 Scientific Equipment and Research Division, Kasetsart University Research and Development Institute, Kasetsart University, Bangkok 10900, Thailand; rdiwnk@ku.ac.th; 3 Department of Microbiology, Faculty of Medicine, Khon Kaen University, Khon Kaen 40002, Thailand; chapie@kku.ac.th (C.P.); tipeka@kku.ac.th (T.E.); 4 HPV & EBV and Carcinogenesis Research Group, Khon Kaen University, Khon Kaen 40002, Thailand

**Keywords:** Stingless bee, honey, propolis, *Tetragonula drescheri*, *Tetragonula pagdeni*, metabolite, human papillomavirus, herpes simplex virus, cervical cancer, soap

## Abstract

Honey and propolis from the stingless bees *Tetragonula drescheri* and *Tetragonula pagdeni* remain underexplored for their health-promoting application. This study investigated the bioactive compounds, and antiviral and anticancer activities of honey and propolis extracts against herpes simplex virus (HSV), and human papillomavirus (HPV-16/18)-positive cervical cancer cells. Water and ethanol extracts were prepared and evaluated for anti-HSV activity using plaque assay, and for anticancer effects on CaSki and HeLa cells using apoptosis, colony formation, cell migration, and candidate gene expression analysis. Propolis water extract most potentially inhibited HSV wild-type and drug-resistant strains. Propolis ethanol extract from *T. drescheri* markedly suppressed CaSki and HeLa cell growth, induced apoptosis, downregulated HPV-16/18 E6, and upregulated *BAX* expression. Chemical profiles were identified by electrospray ionization quadrupole time-of-flight mass spectrometry. Most candidate compounds displayed preferable drug-likeness properties. Prototype herbal soup formulations containing selected extracts significantly inhibited HSV-1 drug-resistant strain and HPV-16 *E6* expression. These findings demonstrated the high antiviral and anticancer potential of the extracted compounds from *T. drescheri* and *T. pagdeni* propolis, supporting their application in health-promoting products against HSV and HPV infection.

## 1. Introduction

Viral infections contribute to roughly one-third of global annual mortality [1]. Human papillomavirus (HPV) and herpes simplex virus (HSV) represent a major group of viruses capable of infecting the skin and mucosal tissues of more than one billion people worldwide [2,3]. In addition, both viruses remain major public health concerns due to their high prevalence, persistent nature, and association with chronic disease and malignancy [2,3].

HPV is recognized as one of the most widespread sexually transmitted pathogens and is linked to the development of warts (condylomas) as well as several malignancies in both men and women, including anus cancer, penis cancer, oropharyngeal cancer, and particularly cervical cancer [4]. More than 100 HPV genotypes have been identified and classified into low-risk and high-risk categories according to their cancer-causing potential. Typically, low-risk HPV types produce benign skin warts, whereas high-risk types are strongly associated with malignancies of the cervix, anus, penis, vagina, vulva, and specific regions of the oral cavity and pharynx [5]. Cervical cancer remains the fourth most prevalent cancer among women worldwide and is the second most common cancer in Thai women [6,7]. HPV-16 and 18 are the most frequently detected, being strongly associated with various malignancies [8]. Generally, the current therapeutic approaches for cervical cancer involve chemotherapy alongside radiotherapy and surgical intervention. Although these therapies are essential for effective cancer control, often they are associated with a range of adverse effects in patients [9].

Herpes simplex virus (HSV) is a highly common sexually transmitted infection responsible for both genital and oral lesions [10]. The virus spreads efficiently through close interpersonal contact. HSV is classified into two serotypes, HSV-1 and HSV-2, which are distinguished by their serological profiles, genetic characteristics, and typical anatomical sites of infection [11]. Typically, HSV-1 infects the oral cavity and adjacent tissues, being most often linked to oral herpes, which manifests as painful, fluid-filled blisters around the lips, mouth, or face. In contrast, HSV-2 predominantly targets the genital tract and is the principal cause of genital herpes. Transmission of either serotype occurs mainly through direct contact with an infected person, particularly during periods of active viral shedding when lesions are present. After primary infection has occurred, HSV establishes a lifelong infection because the virus remains latent within the nerve ganglia and cannot be completely eliminated [12]. Recurrent HSV-associated disease may occur more frequently in both immunocompromised and immunocompetent individuals because of their susceptibility to viral reactivation [10,13]. Although antiviral agents, such as acyclovir, are used widely to treat HSV-associated diseases, long-term particularly in immunocompromised and immunodeficient individuals, can contribute to the emergence of drug-resistant viral strains that can reduce effectiveness of these anti-viral agents [14].

Stingless bees, commonly known as bees without stingers, are unable to sting attackers but defend their colonies by biting. They belong to the family *Apidae* and are closely related to honeybees, carpenter bees, orchid bees, and bumblebees [15,16]. Thailand hosts a greater diversity of stingless bees than honeybees, with 33 species across 10 genera widely distributed throughout the country and locally referred to as “Channarong”. Most species nest in natural tree cavities and feed primarily on nectar and pollen [17,18]. Traditionally, stingless bee’s honey, propolis, pollen, royal jelly, and beeswax have been used for medicinal purposes in Thailand [19]. In recent decades, propolis has gained increasing attention for its applications in medicine and cosmetics, although it has long been recognized in traditional and ancient remedies [20]. Propolis is a resinous material collected by stingless bees from tree sap and plant buds, which is then mixed with bee enzymes, pollen, and wax to seal hive cracks, prevent infections, and maintain colony hygiene. This natural product exhibits multiple pharmacological activities, including antimicrobial, anticancer, anti-inflammatory, and antioxidant properties [21,22]. Stingless bee propolis varies in composition depending on botanical sources, geographical location, and bee species [23]. Typically, it contains approximately 50% resins that are rich in flavonoids and polyphenolic acids, 30% waxes, 10% essential oils, 5% pollen, and 5% other organic compounds [24,25]. Phenolic acids represent the most important bioactive group due to their strong antiviral properties. In addition the honey from stingless bees has recently attracted attention for its medicinal potential, including antioxidant, anti-inflammatory, anti-obesity, anticancer, and antimicrobial activities [26]. However, only a few studies have been published on Thai native stingless bee propolis and honey. Therefore, the current study investigated *Tetragonula drescheri* (Schwarz, 1939) and *Tetragonula pagdeni* (Schwarz, 1939) because both species are reared widely by Thai farmers. Predominantly, *T. drescheri* is found in southern Thailand. *T. pagdeni* is highly adaptable and commonly is found throughout the country. Different stingless bee species exhibit distinct habitat distributions depending on their preferred food, nesting tree species, and the source of resin they collect. These factors may impact on the bioactive compounds and their biological activity in medical applications.

Therefore, the current study investigated the effects of honey and propolis extracts from two different Thailand stingless bee species (*T. drescheri* and *T. pagdeni*) on the antigrowth of HPV-positive cervical cancer cells and anti-infectivity of HPV-16 and wild type HSV, drug-resistant HSV-1 strains, as well as their bioactive compound content, with the goal of developing a prototype model for health promotion products. In addition, molecular docking was performed to analyze possible mechanism and drug-likeness properties of most candidate compounds found in both extracts.

## 2. Results

### 2.1. Bioactive Compounds

Table 1 and Table S1 summarize the concentrations of total polysaccharides, total proteins, tannin content, and DPPH activity of all 14 extracts. The honey-derived extracts and PEE-D yielded the highest polysaccharide concentration. The tannin content was greatest in the ethanol-based extracts, specifically, PEE-D (148.28 ± 3.60 mg/mL) was the highest, followed by PEE-P (46.87 ± 2.03 mg/mL). Among the 14 extracts, PEE-D had the highest levels of protein and tannin, followed by PWE-D_100. Notably, PWE-D_100 (IC_50_ = 0.26 ± 0.00 mg/mL) had the highest DPPH activity, followed by PEE-D (0.70 ± 0.01 mg/mL) and PWE-P_100 (IC_50_ = 0.73 ± 0.01 mg/mL). Based on these results, *T. drescheri* was the more promising source of a bioactive compound as a particular antioxidant agent, followed by *T. pagdeni* propolis.

### 2.2. Effects of Extracts from T. drescheri and T. pagdeni on Cytotoxicity in Vero, CaSki, HeLa, and 293FT Cell Lines

The cytotoxicity of the 14 extracts is shown in Table 2 and Table S2. PEE-D had the strongest cytotoxic effect on the CaSki and HeLa cells, followed by PEE-P. Notably, both PEE-D and PEE-P were more toxic to these cancer cell lines than the noncancerous Vero cell line. In addition, the honey-derived extracts had the lowest cytotoxic effects on all 4 cell lines followed by the water-derived extracts. PEE-D and PEE-P were selected to investigate their effect against growth of CaSki and HeLa cells, whereas all of 14 extracts were further tested for anti-HSV infectivity.

### 2.3. Anti-HPV Infectivity and Effect on Growth of HPV Positive Cervical Cancer Cells

#### 2.3.1. Effects of PEE-D and PEE-P on HPV-16 Pseudovirus Infection

**Figure 1:**
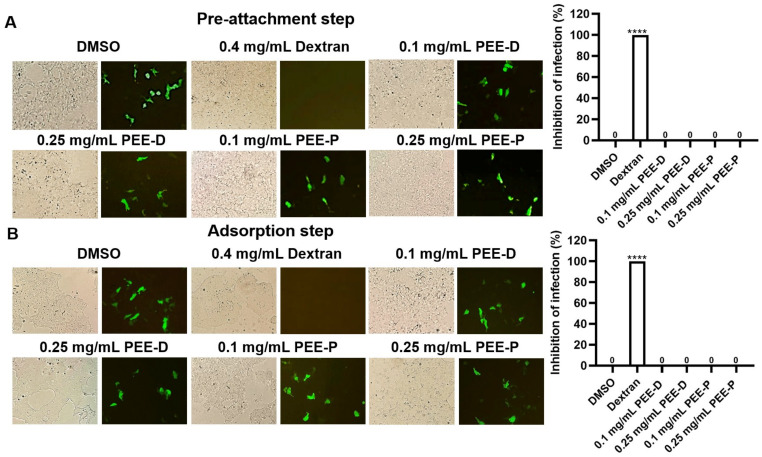
Effect of PEE-D and PEE-P on PsV16-infection assay during (**A**) pre-attachment step and (**B**) adsorption step. The 293FT cells were treated with each extract. Dextran and DMSO were used as positive and negative controls, respectively. The symbol **** indicates significant differences (*p* < 0.0001). Bar charts represent the mean ± SEM of triplicate experiments.

To investigate the ability of PEE-D and PEE-P against HPV infection, HPV-16 Pseudovirus (PsV16) was produced and then tested with these extracts at concentrations of 0.1 and 0.25 mg/mL in the pre-attachment and adsorption steps. The results are shown in Figure 1. Treatment with 0.4 mg/mL dextran (positive control) completely inhibited PsV16. In contrast, neither PEE-D nor PEE-P exhibited any inhibitory activity at either concentration in both the pre-attachment and adsorption steps.

#### 2.3.2. PEE-D and PEE-P Induced Cell Death in HPV-Positive CaSki and HeLa Cells

**Figure 2:**
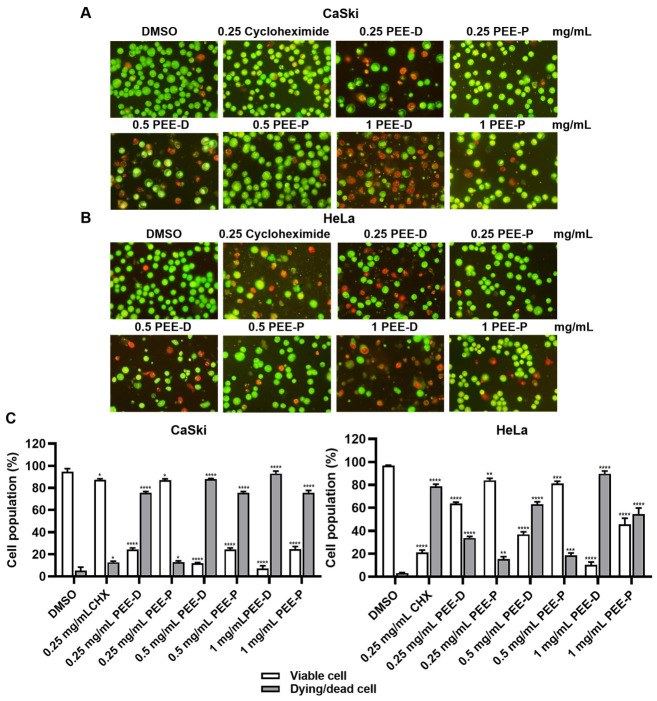
Morphology of PEE-D-treated and PEE-P-treated CaSki and HeLa cells dual-stained with AO/EB staining. (**A**) CaSki and (**B**) HeLa were treated with PEE-D, and PEE-P for 48 h and then dual-stained with AO/EB. (**C**) Percentages of viable cell, dying and dead cells in CaSki and HeLa. The symbols *, **, ***, and **** indicate significant differences (*p* < 0.05, 0.01, 0.001, and 0.0001, respectively). Bar charts represent the mean ± SEM of triplicate experiments.

To compare the effects of PEE-D and PEE-P on cervical cancer cell death using acridine orange and ethidium bromide (AO/EB) staining, they were tested at concentrations of 0.25, 0.5, and 1 mg/mL for 48 h, as shown in Figure 2. Both PEE-D and PEE-P induced dying and dead cells in a dose-dependent manner in the CaSki and HeLa cells. PEE-D was most effective in increasing the percentage of dying/dead cells compared to PEE-P in both cell lines. Overall, PEE-D had a stronger ability to induce dying and cell death than PEE-P at all concentrations tested with the CaSki and HeLa cells.

**Figure 3:**
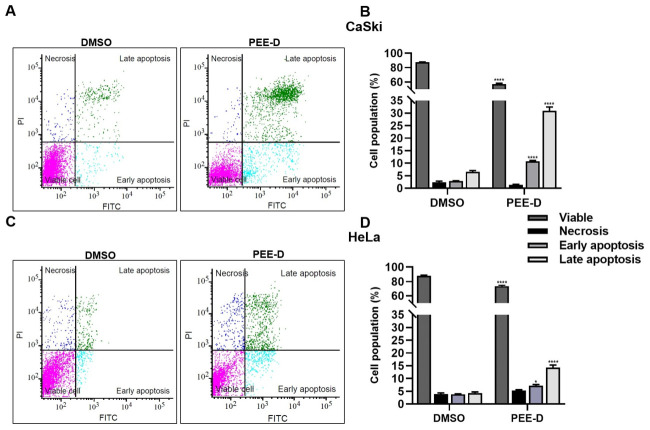
Apoptosis analysis based on flow cytometry in CaSki and HeLa cells, following PEE-D treatment. (**A**,**B**) CaSki and (**C**,**D**) HeLa cells were treated with 0.25 mg/mL PEE-D for 48 h and analyzed using flow cytometry to determine populations of viable, necrotic, early-apoptotic, and late-apoptotic cells. The symbols *, and **** indicate significant differences (*p* < 0.05, and 0.0001, respectively). Bar charts represent the mean ± SEM of triplicate experiments.

To confirm the results from the AO/EB staining, PEE-D at 0.25 mg/mL was investigated essentiality based on flow cytometry. PEE-D at 0.25 mg/mL increased early apoptosis (10.73% ± 0.20% in CaSki and 7.19% ± 0.25% in HeLa) and late apoptosis (30.89% ± 0.90% in CaSki and 14.28% ± 0.57% in HeLa), as shown in Figure 3. Based on these results, PEE-D significantly increased early and late apoptosis in both CaSki and HeLa cells.

#### 2.3.3. PEE-D and PEE-P Reduced Cell Migration and Number of Cancer Colonies

**Figure 4:**
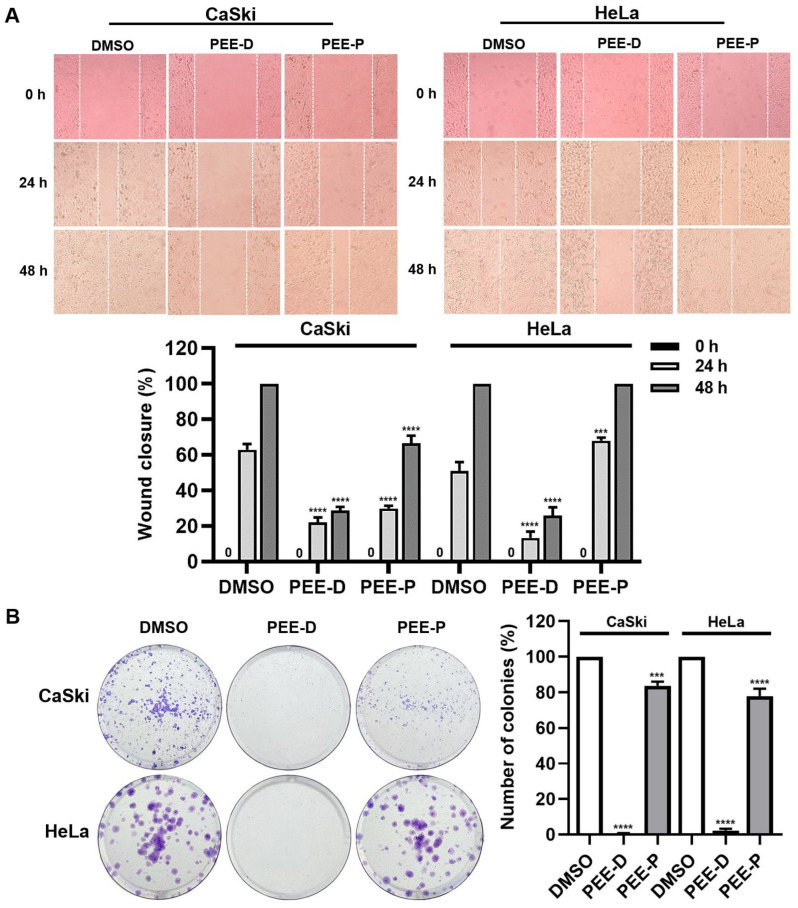
Wound healing and colony formation assays in PEE-D- and PEE-P-treated CaSki and HeLa. (**A**) Wound healing and (**B**) colony formation assays were measured in PEE-D- and PEE-P-treated cells. The percentage of wound closure at 0, 24, and 48 h was calculated using ImageJ software (version 1.54g). The symbols ***, and **** indicate significant differences (*p* < 0.001, and 0.0001, respectively). Bar charts represent the mean ± SEM of triplicate experiments.

Wound healing and colony-forming assays were performed to evaluate the effects of 0.25 mg/mL PEE-D and PEE-P in CaSki and HeLa cells. PEE-D markedly reduced cell migration and the number of cancer colonies in both cell lines compared with the DMSO control (Figure 4), whereas PEE-P significantly reduced cell migration only in CaSki cells (not in HeLa cells), having a high inhibitory effect on colony formation only in CaSki. These findings demonstrated that PEE-D strongly suppressed cell migration and the clonogenic ability of HPV-positive cervical cancer cells.

#### 2.3.4. PEE-D and PEE-P Reduced HPV-16/18 *E6* mRNA and Increased *BAX* mRNA Expression

**Figure 5:**
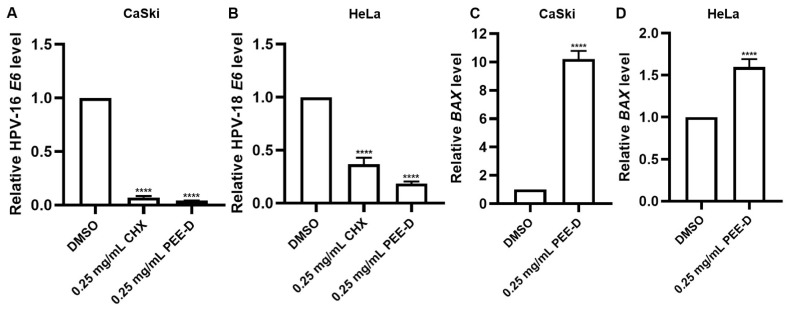
HPV-16/18 *E6* and *BAX* expression in PEE-D-treated CaSki and HeLa. Relative mRNA expression of (**A**) HPV-16 *E6*, (**B**) HPV-18 *E6*, (**C**,**D**) *BAX* in (**A**,**C**) CaSki and (**B**,**D**) HeLa cells was determined using RT-qPCR in PEE-D-treated cells. DMSO and CHX acted as negative and positive controls, respectively. Data are presented as mean ± SEM and were analyzed using one-way ANOVA. The symbol **** indicates significant differences (*p* < 0.0001). Bar charts represent the mean ± SEM of triplicate experiments.

PEE-D at 0.25 mg/mL significantly reduced HPV-16 *E6* and HPV-18 *E6* expression (Figure 5). Notably, PEE-D exerted a stronger suppressive effect on E6 expression than cycloheximide (CHX) in both cell lines. These findings demonstrated that PEE-D inhibited HPV-16/18 *E6* expression resulting in the upregulation of the pro-apoptotic gene *BAX* and consequently reducing the growth of CaSki and HeLa cells.

### 2.4. Anti-HSV Infection

#### 2.4.1. Effect of Honey and Propolis Extracts on HSV-1 KOS, HSV-2 and HSV-1 dxpIII in Pre and Post-Attachment Steps

**Figure 6:**
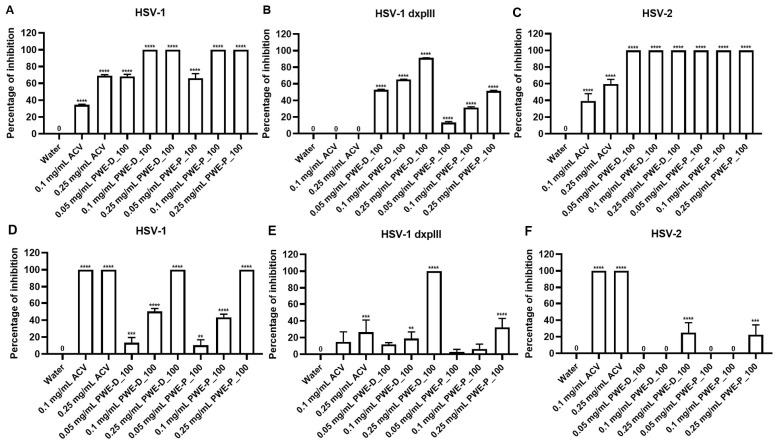
Percentage of inhibition of HSV plaque formation of PWE-D_100 and PWE-P_100-treated Vero cells in pre and post-entry steps. PWE-D_100 and PWE-P_100 were treated to (**A**,**D**) HSV-1 KOS, (**B**,**E**) HSV-1 dxpIII, and (**C**,**F**) HSV-2-infected Vero cells in (**A**–**C**) pre- and (**D**–**F**) post-entry steps. Water and ACV were used as the negative and positive controls, respectively. The symbols **, ***, and **** indicate significant differences (*p* < 0.01, 0.001, and 0.0001, respectively). Bar charts represent the mean ± SEM of triplicate experiments.

Sub-cytotoxic concentrations were used to screen the effect of the 14 extracts against HSV infection. Extracts derived using propolis-based RT, using 50 °C water and honey from *T. drescheri* and *T. pagdeni,* showed low effective inhibition in the post-entry steps of HSV-1; therefore, they were not tested further (Figure 6 and Figure S1). In contrast, water-based propolis extracts at 100 °C and PEE-D showed highly effective inhibition of HSV plaque formation (Figure S1); however, this ethanol-based extract was more cytotoxic to Vero cells than the others (Table 2 and Table S2). PWE-D_100 and PWE-P_100 were investigated further for their effects against HSV-1 dxpIII and HSV-2. Both extracts at 0.25 mg/mL significantly increased the %Inhibition in HSV-1 dxpIII and HSV-2 compared to Water in the pre-entry step (Figure 6A–C and Figure S2). In addition, both PWE-D_100 and PWE-P_100 at 0.1 mg/mL concentration exhibited 100% inhibition of HSV-1 KOS and HSV-2, which was more effective than ACV in the pre-entry step (Figure 6A,C). Notably, PWE-D_100 and PWE-P_100 inhibited plaque formation in all three viral strains more effectively than ACV (Figure 6A–C). Although PWE-D_100 had a higher effective inhibition of HSV plaque formation than PWE-P_100 (Figure 6D–F), it had higher cytotoxicity and a lower SI value (Table 2 and Table 3). Interestingly, PWE-P_100 at 0.25 mg/mL inhibited HSV-1 dxpIII plaque formation with efficacy comparable to that of ACV (Figure 6E). Based on these data, both PWE-D and PWE-P could be high-potential agents against HSV-1 KOS, HSV-2, and drug-resistant HSV-1 dxpIII in both the pre- and post-entry steps.

#### 2.4.2. Host Cell Receptor Binding, Viral Adsorption, Viral Penetration, Time of Addition, Viral Release Assays on HSV-1 dxpIII

**Figure 7:**
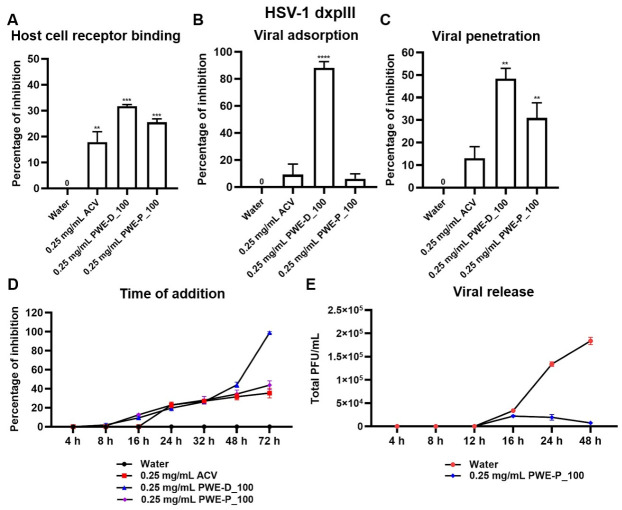
Antiviral effects of PWE-D_100 and PWE-P_100 against HSV-1 dxpIII in host cell receptor binding, viral adsorption, viral penetration, time of addition, viral release assays. PWE-D_100 and PWE-P_100 were tested in Vero cells to investigate their antiviral effects against HSV-1 dxpIII in (**A**) host cell receptor binding, (**B**) viral adsorption, (**C**) viral penetration, (**D**) time of addition (0–72 h), and (**E**) viral release assays. Symbols **, ***, and **** indicate significant differences (*p* < 0.01, 0.001 and 0.0001, respectively) between extract and water-treated cells. Bar charts represent the mean and SEM of triplicate experiments.

Host cell receptor binding, viral adsorption, penetration, and time-additional assays were performed to examine the effects of PWE-D_100 and PWE-P_100 on other mechanism actions against drug-resistant HSV-1 dxpIII. PWE-D_100 significantly increased %Inhibition in the host cell receptor binding, viral adsorption, and penetration stages, while PWE-P_100 could not inhibit the viral adsorption step (Figure 7A–C and Figure S3A–E). Initially, in the time-additional assay, PWE-D_100 and PWE-P_100 inhibited plaque numbers at 16 h of incubation times of the extract, while ACV inhibited plaque formation at 24 h (Figure 7D). As mentioned above, PWE-D_100 caused higher cytotoxicity than PWE-P_100, even though it showed the highest HSV inhibition activity in this assay (Figure 7D and Figure S3D); therefore, PWE-P_100 was investigated further in the viral release step (Figure 7E and Figure S3E). PWE-P_100 at a sub-toxic concentration significantly inhibited the extracellular HSV-1 dxpIII titer (less than 10^4^ PFU/mL).

#### 2.4.3. Effects of PWE-P_100 on mRNA Expression Levels of HSV-1 dxpIII *ICP4*, *UL30*, and *gD* at Different Time Points

**Figure 8:**
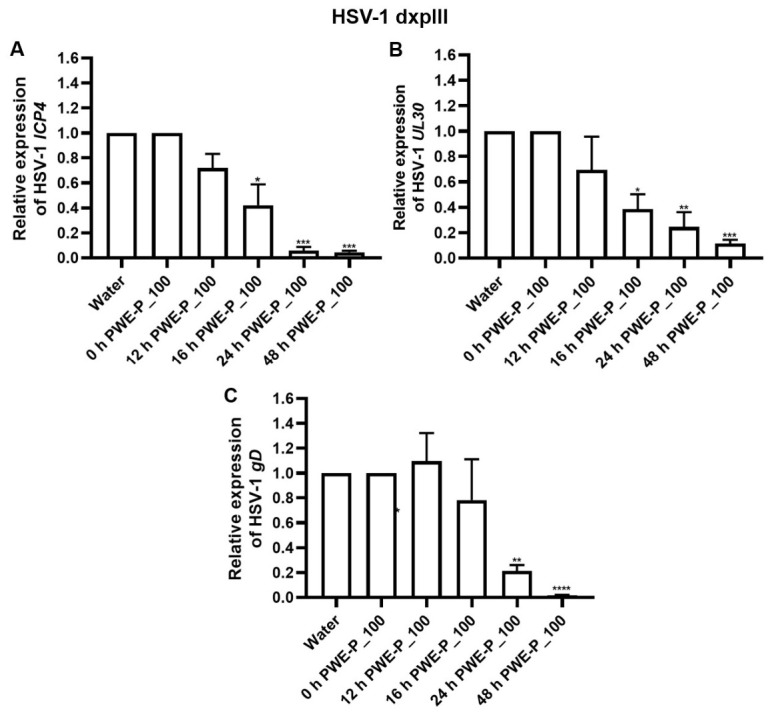
Effect of PWE-D_100 on the expression of HSV-1 dxpIII *ICP4*, *UL30*, and *gD* mRNAs. The mRNA expression levels of HSV-1 dxpIII gens (**A**) *ICP4*, (**B**) *UL30*, and (**C**) *gD* mRNA were analyzed by RT-qPCR. The symbols *, **, ***, and **** indicate significant differences (*p* < 0.05, 0.01, 0.001 and 0.0001, respectively). Bar charts represent the mean ± SEM of triplicate experiments.

The effects of PWE-P_100 on the viral life cycle were evaluated in HSV-1 dxpIII-infected Vero cells. The mRNA expression levels of *ICP4* (immediate early gene), *UL30* (early gene), and *gD* (late gene) were determined after incubation with 0.25 mg/mL PWE-P_100 for 0, 12, 16, 24, and 48 h. PWE-P_100 significantly reduced *ICP4* expression at 16 h (Figure 8A), whereas *UL30* and *gD* expression was significantly decreased at 16 h and 24 h post-infection, respectively (Figure 8B and C). These findings suggested that PWE-P_100 inhibited the expression of immediate early gene *ICP4*, which may have contributed to reducing the mRNA expression of the early and late phases of the viral replication cycle.

### 2.5. Anti-HPV-16 E6 mRNA Expression and Anti-Drug Resistant HSV-1 Activity of PEE-D- and PWE-P_100-Based Prototype Herbal Soaps

**Figure 9:**
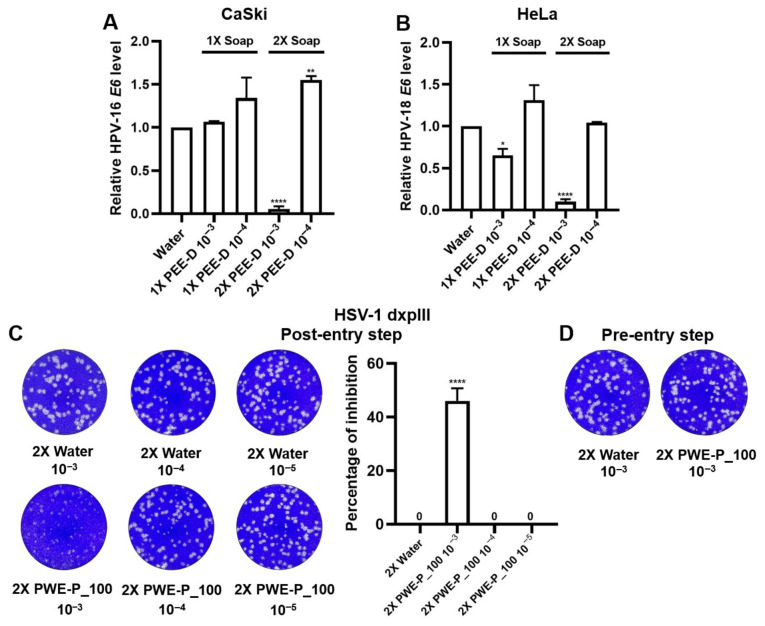
Effect of PEE-D and PWE-P_100 soaps against HPV-16/18 *E6* and HSV-1 dxpIII. (**A**,**B**) HPV-16/18 *E6* mRNA expression in CaSki and HeLa cells after treatment with PEE-D soaps for 48 h were quantified by RT-qPCR. Vero cells infected with HSV-1 dxpIII were treated with 2X PWE-P_100 soaps at 10^−3^–10^−5^ dilutions in the (**C**) post-attachment and (**D**) pre-attachment steps. Plaque formation was visualized by crystal violet staining at 72 h post-infection. The symbols *, **, and **** indicate significant differences at *p* < 0.05, 0.01, and 0.0001, respectively. Bar charts represent the mean ± SEM of triplicate experiments.

A prototype herbal soap consisting of eight formulations was prepared using the two extracts selected as the most suitable (PEE-D and PWE-P_100) (Table S3). Treatment with the 2× PEE-D soap diluted to 10^−3^ (0.25 mg/mL PEE-D) markedly reduced the HPV-16/18 *E6* mRNA levels in the CaSki and HeLa cells compared to the DMSO-control soaps (Figure 9A,B), while the 1× PEE-D soap diluted to 10^−3^ (0.05 mg/mL PEE-D) substantially decreased HPV-18 *E6* mRNA expression (Figure 9B). However, only the 2× PWE-P_100 soap diluted to 10^−3^ (0.25 mg/mL PEE-D) exhibited antiviral activity against the drug-resistant HSV-1 strain (46.02% ± 4.72% of inhibition), as shown in Figure 9 and Figure S4. These data provide insight into the promising application of soaps containing PEE-D and PWE-P_100 against HPV-16/18 *E6* expression and HSV-1 dxpIII infection.

### 2.6. Metabolites in PEE-D and PWE-P_100

**Figure 10:**
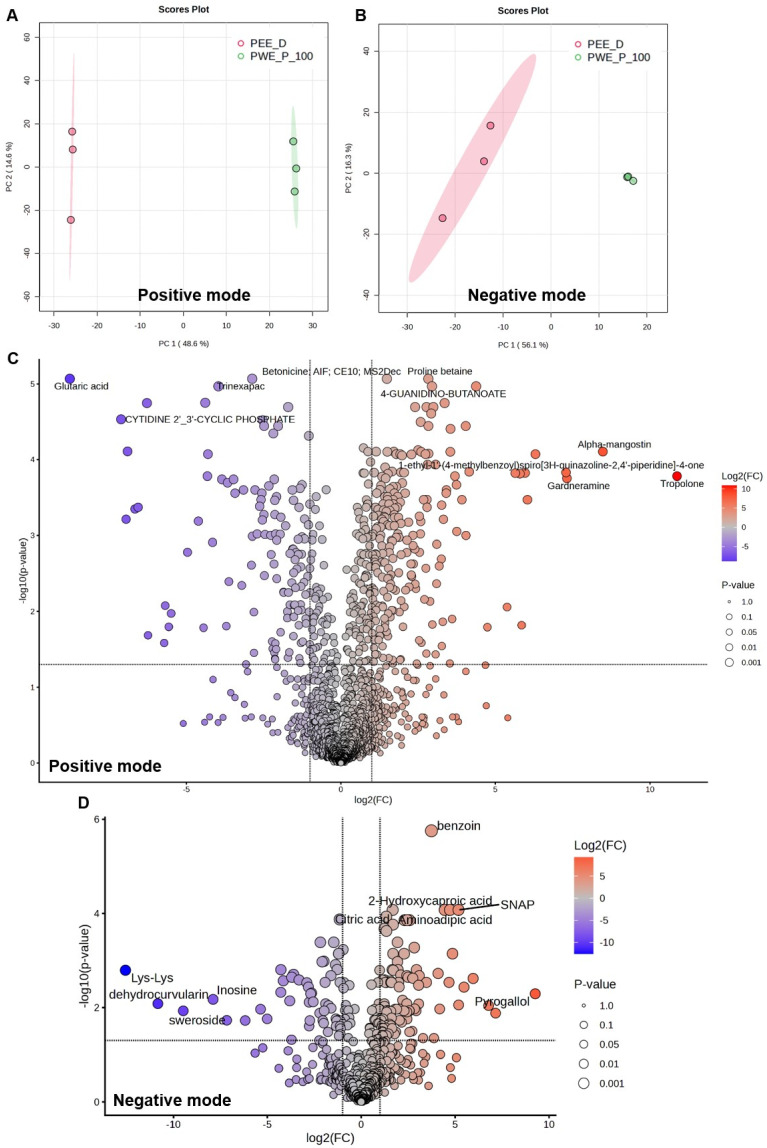
PCA score plots, and volcano plot analysis of the metabolites identified in PEE-D and PWE-P_100. (**A**,**B**) PCA score plot, and (**C**,**D**) volcano plot analysis were analyzed using the web software MetaboAnalyst 6.0 platform. (**C**,**D**) The vertical dashed lines indicate the fold change threshold (log2FC = ±1), while the horizontal dashed lines represent the significant threshold (−log10 *p*-value = 1.3, *p* < 0.05).

**Figure 11:**
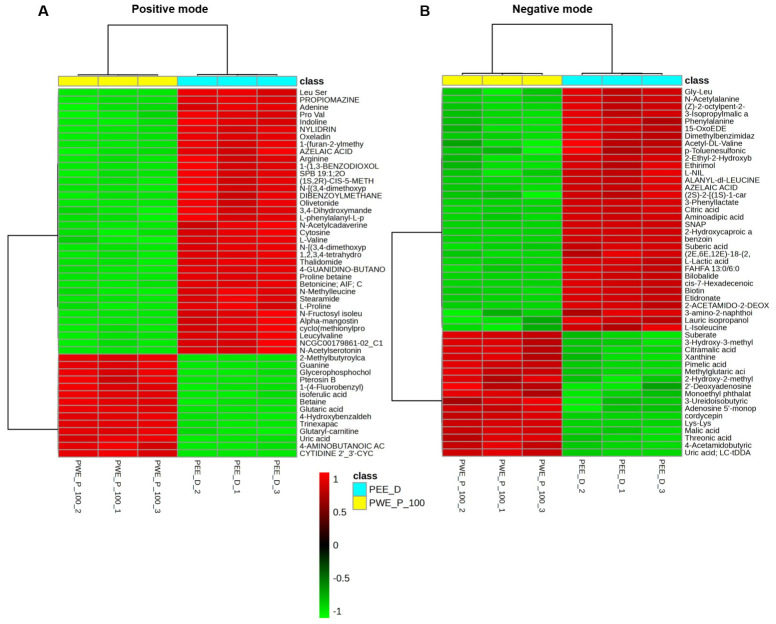
Heatmap of the metabolites identified in PEE-D and PWE-P_100. Hierarchical clustering heatmap of the top 50 significant features (FDR < 0.05) in (**A**) positive and (**B**) negative modes were analyzed by the web software MetaboAnalyst 6.0 platform.

**Figure 12:**
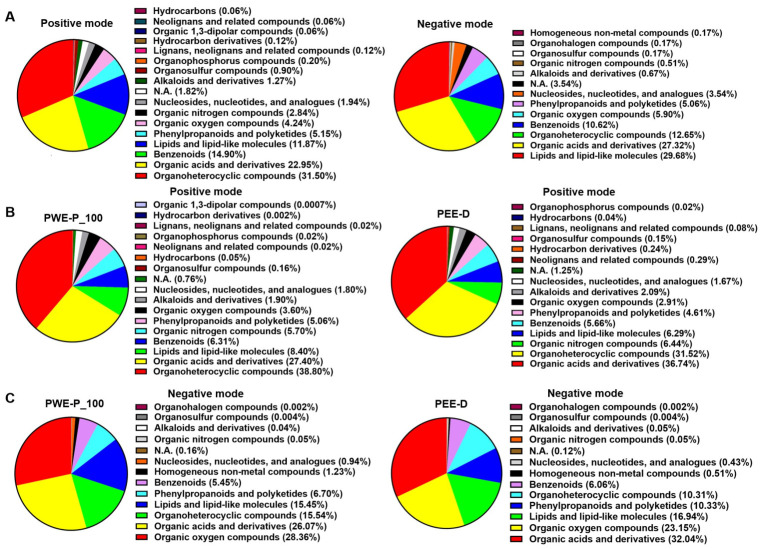
Pie charts showing the distribution of identified metabolites among chemical superclass-based compound counts and total peak areas. (**A**) Pie charts were established based on compound counts in the positive and negative modes. (**B**,**C**) Pie charts were established based on total peak areas in each superclass.

Total 1650 and 598 metabolites of the extracts were identified in positive and negative modes, respectively (Figure S5, Files S1 and S2). Guanine and proline betaine were most abundant in PWE-P_100 and PEE-D in positive mode, whereas gluconic acid, a common compound, was abundant in both extracts in negative mode based on relative total peak area in all metabolites (Table 4). However, the metabolic profiles of both extracts displayed more distinct patterns, based on the principal component analysis (PCA) (Figure 10A,B). Volcano plot analysis identified 488 and 181 significant features (FDR < 0.05) in positive and negative modes, respectively. Two hundred seventy-nine upregulated and 150 downregulated metabolites were found in PEE-D compared with PWE-P_100 in positive mode, whereas 92 upregulated and 52 downregulated metabolites were found in negative mode (Figure 10C,D). A heatmap based on the top 50 significantly altered metabolites (ranked by adjusted *p*-value) demonstrated distinct metabolic profiles between the two extracts (Figure 11A,B). The distribution of identified metabolites based on compound counts is presented in Figure 12A. Based on ClassyFire superclasss classification, the detected metabolites were mainly distributed among organoheterocyclic compounds, organic acids and derivatives, benzenoids, and lipids and lipid-like molecules in the positive mode. In contrast, lipids and lipid-like molecules, and organic acids and derivatives were the predominant superclasses in the negative mode, followed by organoheterocyclic compounds, and benzenoids. Simultaneously, the relative abundance of metabolites based on total peak areas in the positive mode is shown in Figure 12B. In PWE-P, organoheterocyclic compounds represented the most abundant superclass, followed by organic acids and derivatives, whereas organic acids and derivatives were most predominant in PEE-D, followed by organoheterocyclic compounds. In negative mode, organic oxygen compounds, and organic acids and derivatives yielded the highest total peak areas in PWE-P and PEE-D, respectively (Figure 12C). Overall, the compound diversity observed in PEE-D and PWE-P indicates that both extracts contain similar types of chemical superclasses. In addition, the distribution of relative metabolite abundance based on total peak areas differed between PWE-P_100 and PEE-D, suggesting variations in the dominant metabolite groups present in each extract that may influence different biological activities.

## 3. Discussion

In Thailand, stingless bee honey and propolis have been used in traditional medicine; however, their biological activity and safety are strongly influenced by the extraction conditions, including the solvent type, temperature, stingless bee species, and biological source. Differences in extraction methods can markedly affect both the yield of bioactive compounds and the safety profile of natural extracts, corresponding to the present findings [27,28]. Another report suggested that propolis extracted with 60–80% ethanol strongly inhibited microbial growth and provided high antioxidant activity [29]. However, the higher phenolic content in ethanolic extracts may increase cytotoxicity. In contrast, the water-based honey (HWE) and propolis (PWE) extracts in the present study produced lower cytotoxicity than the ethanolic extracts. This observation was consistent with another study that reported the lower cytotoxicity of PWEs than ethanolic extracts, while they showed effective antimicrobial activity, supporting their potential as a safe alternative for medical and health-related applications [30]. In the current study, the strong antioxidant activity observed in the PWE_100 and ethanolic propolis extracts was consistent with their high phenolic contents, supporting the role of phenolic compounds, including flavonoids, as major contributors to DPPH radical scavenging activity. These findings were consistent with another study reporting that stingless bee propolis extracts contained substantial amounts of phenolic compounds responsible for their potent antioxidant properties [31].

Based on the cytotoxicity results in the current study, PEE exhibited stronger suppression of cell viability in HPV-positive cervical cancer cells than PWE. Furthermore, PEE-D had a more pronounced effect than PEE-P. Notably, both PEE-D and PEE-P had higher cytotoxicity than noncancerous Vero cells (Table 2). These findings suggest that ethanol extraction may enrich the bioactive compounds associated with anticancer activity, consistent with previous report indicating that ethanolic propolis extracts contain higher levels of phenolic and flavonoid compounds [25]. In contrast, the lower cytotoxicity of PWE indicated that water-based extracts may be more suitable for antiviral applications rather than cancer-targeted studies.

Although PEE-D and PEE-P did not inhibit HPV-16 pseudovirus infection in the pre-attachment or adsorption steps, they exhibited anticancer activity in HPV-positive cervical cancer cells (Figure 1, Figure 2, Figure 3, Figure 4 and Figure 5). These findings indicated that the anticancer effects of ethanolic propolis extracts were not mediated by blocking viral entry, but rather by interfering with intracellular oncogenic pathways. Both PEE-D and PEE-P significantly increased the populations of dying and dead cells in CaSki and HeLa cells in a dose-dependent manner, with PEE-D exhibiting a stronger pro-apoptotic effect. This observation was consistent with previous study reporting that natural compounds induced cancer cell death predominantly through apoptosis by modulating mitochondrial integrity and regulating pro-apoptotic gene expression [32]. Notably, the HeLa cells were more sensitive to the PEE-D treatment than the CaSki cells, as indicated by a higher proportion of late apoptotic cells, suggesting differential cellular responses related to HPV type, oncogene expression levels, or intrinsic cell-type difference (Figure 3). Corresponding to the apoptosis results, PEE-D markedly suppressed cell migration and colony-forming ability, indicating inhibitory effects on aggressive cancer cell behaviors (Figure 3 and Figure 4). Another study reported that propolis and its bioactive constituents suppressed cancer cell migration and colony-forming ability through the induction of apoptosis and the modulation of survival-related signaling pathways [33]. This suppression may be associated with downregulation of an oncogenic HPV E6 expression. Previous study reported that upregulation of HPV-16 E6 promoted cancer cell migration and colony-forming ability by modulating cadherin signaling and oncogenic transcription factors, whereas inhibition of E6 restored apoptotic signaling and reduced these malignant properties [34]. Consistent with these observations, PEE-D significantly downregulated HPV-16/18 *E6* expression while concurrently upregulating the pro-apoptotic gene *BAX*. The simultaneous decrease in *E6* and increase in *BAX* expression may suggest the restoration of apoptotic signaling in the HPV-positive cervical cancer cells.

All honey and propolis extracts were initially screened for antiviral activity under non-cytotoxic conditions. The HWEs showed low inhibitory activity against HSV infection, consistent with previous reports indicating that stingless bee honey exhibited primarily antibacterial rather than antiviral activity [28]. In contrast, PWE-D_100 and PWE-P_100 exhibited strong antiviral activity against all three HSV strains. The enhanced antiviral activity observed at 100 °C suggested that elevated extraction temperatures may facilitate the release of the bioactive compounds associated with antiviral activity, thereby improving inhibition at the early stages of viral infection. Although these extracts showed lower inhibitory activity against HSV-1 KOS and HSV-2 during the post-attachment step compared with acyclovir, they were more effective against of HSV-1 dxpIII. HSV-1 dxpIII is a phosphonoacetic acid- and phosphonoformate-resistant strain associated with alterations in viral DNA polymerase, resulting in resistance to pyrophosphate analogue antiviral agents [35,36]. Acyclovir, a deoxyguanosine analogue, requires activation by viral thymidine kinase and subsequently inhibits viral DNA polymerase through competitive incorporation into viral DNA [36,37]. Resistance to acyclovir is commonly attributed to mutations in viral thymidine kinase or DNA polymerase, leading to impaired drug activation or reduced binding affinity [38,39]. The effective inhibition of HSV-1 dxpIII by PWE-D_100 and PWE-P_100 suggested that the antiviral mechanisms of propolis water-based extracts differed from those of nucleoside analogues and may remain effective against drug-resistant HSV strains. This is consistent with previous findings showing that herbal extracts suppressed drug-resistant HSV strains more effectively than acyclovir [40].

In addition, propolis water-based extracts interfered with multiple steps of viral infection, including host cell receptor binding, viral adsorption, and penetration, consistent with previous reports describing natural products that targeted various stage of HSV infection [41]. However, PWE-D_100 also exhibited higher cytotoxicity toward host cells. In contrast, PWE-P_100 did not inhibit the viral adsorption step, suggesting that its antiviral activity may occur after initial attachment. Notably, PWE-P_100 significantly reduced extracellular HSV-1 dxpIII titers to below 10^4^ PFU/mL (Figure 7E). Treatment with PWE-P_100 resulted in a significant reduction in the expression of the immediate early gene *ICP4* at 16 h post-infection, the early gene *UL30* and the late gene *gD* at 24 h. Given the central role of *ICP4* in initiating HSV transcription, suppression of this immediate early regulator may contribute to the downstream inhibition of viral DNA replication and late protein synthesis [42]. This coordinated downregulation of viral gene expression was consistent with the observed reduction in plaque formation and extracellular viral release, supporting a multilevel inhibitory effect of PWE-P_100 on the HSV replication cycle.

HSV DNA polymerase and glycoprotein D (*gD*) are targeted by acyclovir and dextran, respectively. HPV-16 E6 plays a central role in cervical carcinogenesis. These proteins were selected to investigate the possible mechanism of candidate compounds found in PWE-P_100 and PEE-D based on molecular docking. Among the abundant compounds identified in the extracts, citrinin, N-(2-methylquinolin-5-yl)cyclopropanecarboxamide, and uric acid exhibited favorable binding energies toward HSV-1 DNA polymerase, HSV-1 gD, and HPV-16 E6, respectively (Figures S6 and S7). These interactions may have contributed to the inhibition of viral replication, viral entry, or oncogene-mediated survival signaling. Nevertheless, these findings from molecular docking results represent only a preliminary analysis of potential interactions with viral target proteins. Due to the limitations of the PyRx platform, a further docking validation study is required to confirm these interactions. In addition, most of the major compounds identified in both PEE-D and PWE-P_100 showed acceptable drug-likeness properties based on SwissADME analysis, supporting their suitability as bioactive components (Table S4). However, citrinin is a nephrotoxic mycotoxin and a major global concern in contaminated food [43,44]. Zargar et al., 2023 demonstrated that the predicted median fatal dosage of this compound was 105 mg/kg weight, which belong to toxicity class 3 when swallowed [44]. Therefore, tropical administration may reduce its toxicity. In addition, they also suggested that the moisture hot temperature reduced the toxicity. Together, these in silico findings supported the experimental observations that PWE-P_100 exerted antiviral activity against HSV, including the drug-resistant HSV-1 dxpIII strain, while PEE-D displayed pronounced anticancer activity through suppression of HPV oncogene expression and induction of apoptosis. Although molecular docking does not provide definitive evidence of in vivo efficacy, the predicted interactions provide supportive mechanistic evidence linking the chemical composition of stingless bee propolis extracts to their biological effects, as commonly applied in structure-based drug discovery studies [45]. Furthermore, PWE-P_100 and PEE-D were incorporated into prototype soap formulations as a proof-of-concept for product development. The formulations showed biological activity by significantly reducing HSV-1 dxpIII infection and HPV-16/18 *E6* mRNA expression, suggesting that both PWE-P_100 and PEE-D could be used to develop health-promoting products such as herbal soap.

The current study has some important limitations. The propolis and honey were purchased from the same stingless bee farm in Narathiwat province, Thailand. The bioactive compound and biological activity may change if the samples were collected from other regions and in different seasons. Using only in vitro and in silico studies cannot provide definitive results regarding the outcomes of these extracts against HSV, HPV, and cervical cancer; therefore, in vivo and ex vivo studies are needed in further investigation. Although the extracts and soap prototypes for health showed the potential antiviral and anticancer activities, some compounds particular citrinin should be further removed or investigated their toxicological risks before practical use.

## 4. Materials and Methods

### 4.1. Cell Culture

CaSki (HPV-16-positive cervical cancer cells) and HeLa (HPV-18-positive cervical cancer cells) cell lines were kindly obtained from Prof. Dr. Tohru Kiyono (National Cancer Center Research Institute, Tokyo, Japan). Human embryonic kidney 293FT were purchased from Invitrogen (Carlsbad, CA, USA). An African green monkey kidney cell line (Vero) kindly provided by Prof. Dr. Pilaipan Puthavathana (Center for Research and Innovation, Faculty of Medical Technology, Mahidol University, Nakhon Pathom, Thailand). The cultivation of all cells was carried out in Dulbecco’s modified eagle medium (DMEM) (Gibco Laboratories, Grand Island, NY, USA) with 10% fetal bovine serum (FBS) (Hyclone, Thermo Scientific, Logan, UT, USA). Gentamycin (40 µg/mL), amphotericin B (2.5 µg/mL), streptomycin (100 µg/mL), and penicillin G (100 units/mL) were added to the medium before use. The cell cultures were incubated at 37 °C with 5% CO_2_.

### 4.2. Viruses

HSV-1 KOS and HSV-2 were acyclovir-susceptible strains (kindly provided by Prof. Dr. Pilaipan Puthavathana, Center for Research and Innovation, Faculty of Medical Technology, Mahidol University, Nakhon Pathom, Thailand). HSV-1 dxpIII was a phosphonoacetic acid- and phosphonoformate-resistant variant (kindly provided by Prof. Dr. Donald Coen, Biological Chemistry & Molecular Pharmacology, Harvard Medical School, Boston, MA, USA). All viral strains were propagated and titered using plaque assay in Vero cells. Virus stocks were harvested and stored at −80 °C until use.

### 4.3. Extraction of Propolis and Honey

**Figure 13:**
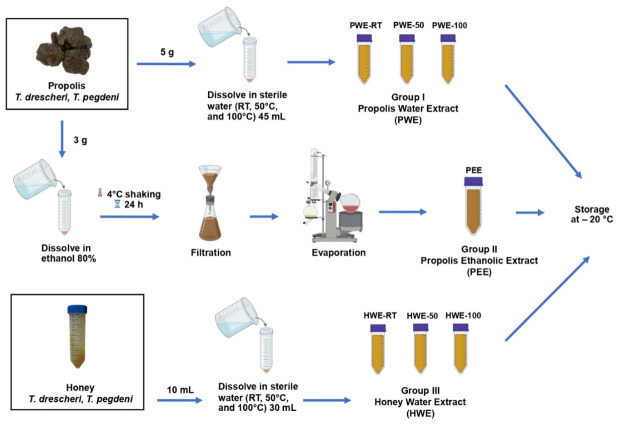
Preparation of propolis and honey extracts from *T. drescheri* and *T. pegdeni*, including water extracts at different temperatures (PWE and HWE) and ethanolic extract (PEE).

The propolis and honey of *T. drescheri* (-D) and *T. pagdeni* (-P) were purchased from Narathiwat province, Thailand, in November 2022. For water-based extraction of propolis (PW), each 5 g sample of the propolis was dissolved in 45 mL of sterile water at different temperatures (room temperature, 50 °C, 100 °C) for 30 min (a stock 100 mg/mL). Another group of propolis samples (3 g each) was dissolved in 27 mL of 80% ethanol and shaken overnight at 4 °C, followed by filtration using Whatman paper No. 4 to remove wax and other insoluble components. Subsequently, the solution was evaporated under reduced pressure using a rotary evaporator to achieve extract dryness and dissolved in Dimethyl sulfoxide (DMSO) to prepare a stock 500 mg/mL. The honey samples (10 mL each) were dissolved in 30 mL of sterile water at different temperatures (room temperature, 50 °C, 100 °C) for 30 min (a stock 250 mg/mL). The extracts were stored at −20 °C until analysis. A schematic diagram of each extract preparation is summarized in Figure 13.

### 4.4. Measurement of Bioactive Compounds

#### 4.4.1. Total Protein

The total protein content in the honey and propolis extracts was determined using the Quick Start™ Bradford Protein Assay (Bio-Rad Laboratories, Hercules, CA, USA) modified from Anjos et al., 2022 [46]. Each extract at 20 µL was subjected in 200 µL of 1× Bradford reagent per well. The absorbance was then measured at 595 nm using a Multiskan™ GO microplate photometer (Thermo Scientific™, Vantaa, Finland). The standard curve of absorbance and concentrations 0, 0.1, 0.25, 0.5, 0.8, and 1 mg/mL of bovine serum albumin (Sigma-Aldrich, St. Louis, Mo, USA) were established.

#### 4.4.2. Total Polysaccharide

The total polysaccharide content in each extract was determined using a phenol/H_2_SO_4_ method modified from Chen et al., 2024 [47]. Each extract at 20 µL was mixed with 20 µL of 5% phenol solution (Sigma-Aldrich, St. Louis, Mo, USA) and then were subjected with 100 µL of H_2_SO_4_ solution (Emsure^®^, Merck, Darmstadt, Germany). The reaction was incubated at room temperature for 10 min in the dark. The absorbance was measured at a wavelength of 540 nm using a Multiskan™ GO microplate photometer (Thermo Scientific™). The standard curve was established from the absorbent values and concentrations at 0, 0.1, 0.25, 0.5, 1, 2.5, 5, 25, 50, and 100 mg/mL of dextran (Sigma-Aldrich, St. Louis, Mo, USA).

#### 4.4.3. Total Tannin Content 

Tannin in each extract was determined using a modified vanillin–sulfuric acid assay modified from Ghalem et al., 2014 [48]. Each extract (60 µL) was added to 300 µL of reaction solution containing 0.1 mg/mL vanillin in 70% H_2_SO_4_ (*v*/*v*) and then incubated at room temperature for 20 min in the dark. The absorbance was measured at a wavelength of 500 nm using a Multiskan™ GO microplate photometer (Thermo Scientific™). The standard curve was established from the absorbent values and concentrations at 0, 0.05, 0.1, 0.25, 0.5, 1, 5, 10, and 50 mg/mL of tannin (LD Carlson, Kent, OH, USA).

#### 4.4.4. Antioxidant Assay Using 2,2-Diphenyl-1-(2,4,6-Trinitrophenyl)hydrazyl (DPPH) Radical Scavenging Capacity

The free radical scavenging activity was evaluating using DPPH [49]. A 0.1 mM DPPH solution was prepared in absolute ethanol. Extract samples at different concentrations in 500 μL absolute ethanol were mixed with 500 μL 0.1 mM DPPH solution (Sigma-Aldrich; St. Louis, MO, USA) and then incubated at room temperature for 30 min using dark conditions. The absorbance was measured at 517 nm using a microplate reader (Sigma-Aldrich, St. Louis, MO, USA). The DPPH activity was calculated based on the formula: %Inhibition = [(Ac − As)/Ac] × 100, where As is the absorbance of the sample with the DPPH solution and Ac is the absorbance of the control reaction which contains all reagents except the sample. The half maximal inhibitory concentration (IC_50_) values were determined based on linear regression analysis and derived from plotting the percentage inhibition of DPPH by the sample (each extract) against concentration. Subsequently, replacing the coefficient y with a value of 50 gives the coefficient x in the linear regression equation, which represents the IC_50_ value. Ascorbic acid (Sigma-Aldrich, St. Louis, MO, USA) was used as the positive control.

### 4.5. Cytotoxicity by MTT Assay

Each Vero, 293FT, CaSki, and HeLa cells were seeded in 96-well plates at a density of 1 × 10^4^ cells/well in DMEM supplemented with 10% FBS and maintained at 37 °C in 5% CO_2_ for 24 h. Cells were treated with various concentrations of propolis and honey extracts and incubated for 48–96 h. Ten microliter of 5 mg/mL MTT (Invitrogen, Carlsbad, CA, USA) was subjected to each well and incubated for 2 h. The formazan was dissolved in DMSO after removal the medium. The absorbance was then measured at 540 nm using a microplate spectrophotometer (Thermo Scientific™). Cell viability was expressed as a percentage relative to untreated controls using the following Equation (1):

%Cell viability = (OD sample/OD control) × 100(1)

### 4.6. Anti-HPV-16 Pseudovirus Infection

#### 4.6.1. Production of HPV-16 Pseudovirus

HPV-16 pseudovirus (PsV16) was produced in 293FT cells co-transfected with pfwB (reporter plasmid) and p16SheLL (encoding HPV-16 L1 and L2) (kindly provided by Prof. Dr. John T. Schiller, Laboratory of Cellular Oncology, Bethesda, MD, USA) using Lipofectamine 2000 (Invitrogen, Carlsbad, CA, USA). The 293FT cells (3720 cells/well) were seeded in 96-well plates and cultured for 4 days at 37 °C in 5% CO_2_. Transfection reactions were assembled with pfwB, p16SheLL, Lipofectamine, and Opti-MEM (Gibco, Carlsbad, CA, USA) following the supplier’s instructions, applied to the cells for 6 h, and then replaced with fresh DMEM for an additional 48 h of culture. Transfected cells were collected, washed with PBS containing 9.5 mM MgCl_2_, and centrifuged at 200× *g* for 5 min at 4 °C. Pellets were resuspended in lysis buffer (PBS with 9.5 mM MgCl_2_, 0.05% Brij 58, and 0.01% RNase) and incubated for 24 h at 37 °C. Viral particles were placed on ice for 5 min and titrated by infecting 293FT cells with serial 10-fold dilutions. GFP expression in infected cells was examined by fluorescence microscopy after 48 h [50].

#### 4.6.2. Antiviral Infection Pre-Attachment Step

To investigate the effect of extracts on PsV16 binding, sub-toxic concentrations of extracts were mixed with PsV16 (MOI 0.1) and incubated for 1 h at 37 °C. The mixtures were then added to 293FT cells for 4 h, after which unbound virus was removed and cells were maintained in complete medium for 48 h. Cells were washed with PBS, harvested, and counted under light and fluorescence microscopy (Olympus BX51, Olympus, Tokyo, Japan). Dextran (4 µM) served as a positive control. Subsequently, the percentage of inhibition was calculated from 100 subtracted by the percentage of infection [50].

#### 4.6.3. Antiviral Infection Adsorption Step

To allow PsV16 to bind to the receptors of host cells without entering the cells, the virus was incubated with 293FT cells at 20 °C for 2 h. Unbound PsV16 was removed, and cells were washed with cold PBS before being cultured in fresh medium with or without extracts for 48 h at 37 °C in 5% CO_2_. The percentage of pseudoviral infection was calculated from the number of infected cells relative to the total cells counted. Subsequently, the percentage of inhibition was calculated from 100 subtracted by the percentage of infection [50].

### 4.7. Cell Death Assay by Acridine Orange and Ethidium Bromide (AO/EB) Staining

CaSki and HeLa cells were seeded in 12-well plates at 1.2 × 10^5^ cells/well and cultured for 24 h at 37 °C in 5% CO_2_. Cells were then treated with different concentrations of propolis extract and incubated for 48 h. Following treatment, cells were collected by trypsinization, centrifuged at 1500 rpm for 5 min at 4 °C, and resuspended in 30 μL of complete medium. A mixture of acridine orange and ethidium bromide (5 μL) was added to the suspension, and stained cells were examined using a hemocytometer under a fluorescence microscope (Olympus BX51) [50]. Cycloheximide (Sigma-Aldrich, St. Louis, MO, USA) acts as a positive control.

### 4.8. Apoptosis Assay Using Flow Cytometry

Apoptotic population of untreated- and treated-CaSki and HeLa cells was determined using an Annexin V-FITC Apoptosis Detection Kit (Dojindo EU GmbH, Munich, Germany) according to the manufacturer’s instructions. Cells were collected, washed twice with PBS, and resuspended in Annexin V binding solution. Subsequently, 5 μL of FITC-conjugated Annexin V and 5 μL of propidium iodide (PI) solution were added to each sample. The mixtures were incubated for 15 min at room temperature in the dark and kept on ice until analysis. Fluorescence signals were measured using a BD FACSMelody™ BRYG 8-Color Cell Sorter (Becton Dickinson; Franklin Lakes, NJ, USA).

### 4.9. Cell Migration by Wound Healing Assay

CaSki and HeLa cells were seeded in 24-well plates at 1 × 10^5^ cells/well and cultured for 24 h at 37 °C in 5% CO_2_. Cells were scratched with a sterile 200 μL pipette tip, followed by treatment with extracts at sub-cytotoxic concentrations for 48 h. Images of wound closure were taken at 0, 24, and 48 h using an inverted microscope (Nikon Eclipse TS100, Tokyo, Japan), and wound gaps were quantified with ImageJ software (version 1.54g, National Institutes of Health, Bethesda, MD, USA) [51].

### 4.10. Colony-Forming Assay

The cells were seeded into 12-well plates at 300 cells/well and maintained for 24 h at 37 °C in 5% CO_2_. Following treatment with the extract for 48 h, the medium was replaced with fresh complete medium and the cells were cultured for an additional 5 days. Colonies were fixed using an acetic acid-to-methanol ration of 3:1 (*v*/*v*), stained with crystal violet, and counted to determine relative colony numbers [51].

### 4.11. Anti-HSV Activities

Antiviral infection of the honey and propolis extracts from *T. drescheri* and *T. pagdeni* was assessed using a plaque assay. The mechanisms of the extracts were determined in different stages of the viral infection, as shown in Figure S8.

#### 4.11.1. Cell CultureAnti-HSV in Pre-Attachment Step

Multiplicity of infection (MOI) at 0.002 of HSV was pre-incubated with various concentrations of the extracts in serum-free medium at 37 °C for 1 h. Subsequently, the mixtures were added to Vero cells and incubated for 2 h. After the unbound virus had been removed by washing with PBS, cells were overlaid with complete medium containing 0.5% carboxymethyl cellulose (CMC) (Sigma-Aldrich, St. Louis, MO, USA) and incubated for 48–72 h. Finally, cells were fixed with 10% formaldehyde and stained with 0.5% crystal violet [40]. Plaques were counted to calculate the percentage of viral inhibition using the equations:

(2) $$\%\mathrm{Infection}=\frac{\mathrm{Number of plaques of treated cells}}{\mathrm{Number of plaques of control cells}}\times 100$$

%Inhibition = 100 − %Infection(3)

#### 4.11.2. Anti-HSV Infection in Post-Entry Step

HSV (MOI 0.001) was infected in Vero cells for 2 h. After removing any unbound virus, the complete medium (containing 0.5% CMC and the various concentrations of the extracts) was treated to the cells for 48–72 h. Then, the cells were fixed with 10% formaldehyde and stained with 0.5% crystal violet [40]. Plaques were counted to calculate the percentage of viral inhibition, as mentioned above.

#### 4.11.3. Viral Binding Assay, Viral Adsorption Assay, Viral Penetration Assay, Viral Release Assay

Vero cells at 1 × 10^5^ cells/well were seeded and incubated for 24 h before performing host cell receptor binding, viral adsorption, penetration, release, and time-of-addition assays using HSV at MOI 0.002 according to Figure S8. After 72 h incubation, plaques were visualized by formaldehyde (Ajax Finechem, UNIVAR, Victoria, Australia) fixation and crystal violet (Merck, Darmstadt, Germany) staining. Plaques were counted to calculate the percentage of viral inhibition, as mentioned above [40].

### 4.12. RNA and Protein Extraction

Vero cells (post-entry assay) and CaSki and HeLa cells (1 × 10^5^ cells/well, 24-well plates) were incubated for 24 h, treated with the extracts, and cultured for an additional 48 h. Cells were subsequently harvested for RNA using TRIzol™ reagent (Thermo Scientific, Waltham, MA, USA) according to the manufacturer’s protocol. For RNA extraction, chloroform (Merck, Darmstadt, Germany) was added, followed by centrifugation for phase separation. The aqueous phase was precipitated with isopropanol, and the RNA pellet was dissolved in nuclease-free water. RNA purity and concentration were determined at 260/280 nm using a Nanophotometer NP80 (Implen, Munich, Germany), and samples were stored at −80 °C.

### 4.13. Determination of Gene Expression Using Real-Time PCR (RT-PCR)

Total RNA was reverse-transcribed into cDNA using the TIANScriptII RT Kit (TIANGEN Biotech (Beijing) Co., Ltd., Beijing, China). Diluted cDNA (1:10) was amplified by real-time PCR on an RqTOWERiris system (Analytik Jena AG, Jena, Germany) with SsoAdvanced SYBR Green Supermix (Bio-Rad Laboratories, Hercules, CA, USA). Each reaction contained 0.3 mM primers (Table S5) and 2 µL of diluted cDNA. The reactions were performed with an initial denaturation at 95 °C for 120 s, followed by 45 cycles of denaturation at 95 °C for 15 s and annealing/extension at 60 °C for 30 s. *GAPDH* was used as the housekeeping gene, and relative expression levels were calculated by the 2^–ΔΔCt^ method compared with untreated controls.

### 4.14. Prototype Herbal Soap Formulations

Prototype herbal soap formulations were prepared using the ingredients listed in Table S3. The 1X and 2X formulations contained 10% and 50% (*v*/*v*) of the selected extract, respectively. A skin-compatible product was obtained by adjusting the pH of each formulation to 5.5–6.5 using citric acid. All components were mixed thoroughly to ensure a uniform mixture, with vigorous stirring being avoided to minimize foam formation. After being allowed to equilibrate at room temperature, the formulations were used directly for antiviral and anticancer assays.

### 4.15. Determination of Chemical Compounds Using Electrospray Ionization Quadrupole Time-of-Flight Mass Spectrometry (ESI-Q-TOF-MS) Analysis

Determination of chemical compounds using ESI-Q-TOF-MS is described in Figure S5. Briefly, the candidate extract powder was resuspended with 0.1% formic acid (LC-MS grade water; CHROMASOLV^®^, Honeywell, Seelze, Germany), filtered and prepared for analysis. Chemical composition analysis was performed using ESI-QTOF-MS system. The separation was carried out by DIONEX Ultimate 3000 HPLC (Dionex Softron GmbH, Germering, Germany) equipped in an Acclaim Advantage II C18 (2.1 × 100 mm, 3 μm) and Acclaim Advantage II C18 (3 × 10 mm, 5 μm) under gradient elution conditions with mobile phases A (water with 0.1% formic acid) and B (acetonitrile with 0.1% formic acid). MSs were acquired in both positive and negative ion modes over a broad *m*/*z* range. Sodium formate was used for external calibration. Data processing, feature extraction, and metabolite identification were conducted using MetaboScape^®^ 2022 software (Bruker, Germany), Bruker MetaboBase Personal Library 2.0 and the MassBank of North America (MONA) database. Only significant metabolites (*p* < 0.05) registered in the Bruker MetaboBase Personal Library 2.0 and MONA databases were included in the final dataset. The ESI-QTOF-MS chromatograms of the candidate extract are presented in Figure S5. The identified metabolites are listed in Files S1 and S2.

### 4.16. Metabolic Profile Analysis

Metabolic profile analysis, including PCA score plots (accessed on 23 February 2026), volcano plot analysis (accessed on 23 February 2026), and heat map visualization (accessed on 24 February 2026), was performed using Metaboanalyst 6.0 web-based platform (https://www.metaboanalyst.ca/) [52]. Chemical classification of the identified metabolites based on superclass was performed using R software (version 4.5.2). The superclass information was retrieved from the ClassyFire database (http://classyfire.wishartlab.com/entities/, accessed on 1–3 March 2026) using InChikey obtained from database including PubChem and ChemSpider (https://www.chemspider.com/search, accessed on 1–3 March 2026).

### 4.17. Statistical Analysis

The results are expressed as mean ± SEM. All experiments were performed in three independent replicates. One-way ANOVA, two-way ANOVA, and the Mann–Whitney U test were used to calculate *p*-values. The *p*-values > 0.05 were considered not significant (“ns”), *p*-values <0.05 were marked as *, <0.01 as **, <0.001 as ***, and <0.0001 as ****, respectively, using GraphPad Prism software (version 8.0.2; GraphPad Software Inc., La Jolla, CA, USA).

## 5. Conclusions

This study showed that the bioactivities of stingless bee propolis extracts from *T. drescheri* and *T. pagdeni* were highly dependent on the extraction conditions. High temperature water-based extracts, particularly PWE-P_100, showed outstanding anti-HSV activity, notably against the drug-resistant HSV-1 dxpIII strain, with minimal cytotoxicity. In contrast, ethanolic extracts, especially PEE-D, displayed potent anticancer effects in HPV-positive cervical cancer cells by inducing apoptosis, suppressing cell migration and clonogenicity, as well as downregulating oncogenic *E6* expression. Molecular docking and drug-likeness analyses provided supportive mechanistic insight into the potential interactions of these extracts with key viral and oncogenic targets. Notably, the incorporation of selected extracts into prototype soap formulations preserved their biological activity. Although further in vivo validation and formulation optimization are required, these findings highlighted the potential of stingless bee propolis extracts as promising alternative or adjunctive agents for managing HSV- and HPV-associated conditions and as promising raw materials for health-promoting functional products.

## Figures and Tables

**Table 1:** Concentration of bioactive compounds and anti-oxidation activity in stingless bee propolis across four extracts.

Extract	Polysaccharide (mg/mL)	Protein (mg/mL)	Tannin Content (mg/mL)	DPPH Activity IC_50_ (mg/mL)
PWE-D_100	178.04 ± 17.13 ^a^	8.0 ± 1.16 ^a^	58.80 ± 2.07 ^a^	0.26 ± 0.00 ^a^
PEE-D	445.12 ± 4.62 ^b^	19.84 ± 2.36 ^b^	148.28 ± 3.60 ^b^	0.70 ± 0.01 ^b^
PWE-P_100	42.61 ± 1.04 ^c^	1.54 ± 0.09 ^c^	2.93 ± 0.10 ^c^	0.73 ± 0.01 ^b^
PEE-P	60.34 ± 0.73 ^d^	5.49 ± 0.27 ^a^	46.87 ± 2.03 ^d^	3.16 ± 0.07 ^c^

Note: PWE-D_100, propolis from *T. drescheri* obtained by water extraction at 100 °C; PEE-D, propolis from *T. drescheri* obtained by ethanol extraction; PWE-P_100, propolis from *T. pagdeni* obtained by water extraction at 100 °C; and PEE-P, propolis from *T. pagdeni* obtained by ethanol extraction. Different lowercase letters (a–d) indicate significant differences among these 4 extracts.

**Table 2:** CC_50_ values (mean ± SEM) of and propolis (PWE and PEE) extracts from *T. drescheri* and *T. pagdeni* in Vero, 293FT, CaSki, and HeLa cells.

Extract	Incubation Time (Hours)	50% Cytotoxic Concentration (CC_50_), mg/mL			
		Vero	293FT	CaSki	HeLa
PWE-D_100	48	0.44 ± 0.01	>0.5	>0.5	>0.5
PEE-D	48	0.46 ± 0.05	0.27 ± 0.01	0.41 ± 0.00	0.30± 0.01
PWE-P_100	48	3.47 ± 0.06	0.45 ± 0.01	>0.5	>0.5
PEE-P	48	1.52 ± 0.03	0.31 ± 0.05	0.99 ± 0.02	0.75 ± 0.02

**Table 3:** IC_50_ (mean ± SEM) and SI values of ACV, PEE-D, PEE-P, PWE-D_100, and PWE-P_100 on anti-HSV.

Extract	HSV-1		HSV-1 dxpIII		HSV-2	
	IC_50_ (μg/mL)	SI (Unitless)	IC_50_ (μg/mL)	SI (Unitless)	IC_50_ (μg/mL)	SI (Unitless)
ACV	0.14 ± 0.01	68.84	429.18 ± 51.83	0.02	0.30 ± 0.00	32.16
PEE-D	75.57 ± 7.24	6.04	N/A	N/A	N/A	N/A
PEE-P	143.04 ± 38.76	10.65	N/A	N/A	N/A	N/A
PWE-D_100	100.38 ± 5.51	4.44	152.70 ± 6.50	2.91	343.23 ± 10.00	1.29
PWE-P_100	117.29 ± 4.42	29.64	185.11 ± 25.21	18.75	422.60 ± 25.57	8.20

N/A denotes “Not available”.

**Table 4:** The six most abundant metabolites identified in PEE-D and PWE-P_100 using electrospray ionization–quadrupole time-of-flight mass spectrometry.

**Positive Mode**					
**Extract**	**Compound**	**CID**	**RT (min)**	***m*** **/*z***	**Mean Peak Area**
PEE-D	Proline betaine (C_7_H_13_NO_2_)	7016562	3.02	144.1017	492,478.67
	N-(2-methylquinolin-5-yl)cyclopropanecarboxamide (C_14_H_14_N_2_O)	652673	10.49	227.1758	462,248.67
	Phenylalanine (C_9_H_11_NO_2_)	6140	9.20	166.0859	423,505.33
	Adenine (C_5_H_5_N_5_)	190	3.92	136.0616	419,311.33
	Choline (C_5_H_13_NO)	305	2.6	104.1066	388,431.33
PWE-P_100	Guanine (C_5_H_5_N_5_O)	135398634	3.98	152.0567	425,295.33
	N-(2-methylquinolin-5-yl)cyclopropanecarboxamide (C_14_H_14_N_2_O)	652673	10.49	227.1758	405,798.00
	Betaine (C_5_H_11_NO_2_)	115244	2.7	118.0857	379,390.67
	Indole-3-ethanol (C_10_H_11_NO)	10685	2.98	162.1112	311,620.67
	Phenylalanine (C_9_H_11_NO_2_)	6140	9.2	166.0859	257,041.33
**Negative Mode**					
**Extract**	**Compound**	**CID**	**RT (min)**	***m*** **/*z***	**Mean Peak Area**
PEE-D	Gluconic acid (C_6_H_12_O_7_)	10690	2.69	195.14457	1,134,958.00
	2-Hydroxycaproic acid (C_6_H_12_O_3_)	99824	12.00	131.13527	343,838.33
	Citric acid (C_6_H_8_O_7_)	311	3.20	191.11129	343,786.00
	L-Lactic acid (C_3_H_6_O_3_)	107689	4.25	89.06814	303,851.33
	Pipecolic acid (C_6_H_11_NO_2_)	849	5.50	128.09754	284,386.00
PWE-P_100	Gluconic acid (C_6_H_12_O_7_)	10690	2.69	195.14457	1,078,842
	Citrinin (C_13_H_14_O_5_)	54680783	0.31	249.08227	205,502.33
	Hydroxyphenyllactic acid (C_9_H_10_O_4_)	9877544	2.68	181.1566	198,860.00
	L-Valine (C_5_H_11_NO_2_)	6287	22.88	115.978	189,209.67
	Uric acid (C_5_H_4_N_4_O_3_)	1175	5.01	167.1023	187,004.67

## Data Availability

The data are available upon reasonable request from the corresponding author.

## Supplementary Materials

The following supporting information can be downloaded at: https://www.mdpi.com/article/10.3390/ijms27093855/s1, refs. [53,54].

## Abbreviations

The following abbreviations are used in this manuscript:

CC_50_	The 50% cytotoxic concentration
ESI-QTOF-MS	Electrospray ionization–quadrupole time-of-flight mass spectrometry
HPV	Human papillomavirus
HSV	Herpes simplex virus
IC_50_	The half maximal inhibitory concentration
MOI	Multiplicity of infection
HWE-D_RT	Honey from *T. drescheri* obtained by water extraction at room temperature
HWE-D_50	Honey from *T. drescheri* obtained by water extraction at 50 °C
HWE-D_100	Honey from *T. drescheri* obtained by water extraction at 100 °C
HWE-P_RT	Honey from *T. pagdeni* obtained by water extraction at room temperature
HWE-P_50	Honey from *T. pagdeni* obtained by water extraction at 50 °C
HWE-P_100	Honey from *T. pagdeni* obtained by water extraction at 100 °C
PWE-D_RT	Propolis from *T. drescheri* obtained by water extraction at room temperature
PWE-D_50	Propolis from *T. drescheri* obtained by water extraction at 50 °C
PWE-D_100	Propolis from *T. drescheri* obtained by water extraction at 100 °C
PWE-P_RT	Propolis from *T. pagdeni* obtained by water extraction at room temperature
PWE-P_50	Propolis from *T. pagdeni* obtained by water extraction at 50 °C
PWE-P_100	Propolis from *T. pagdeni* obtained by water extraction at 100 °C
PEE-D	Propolis from *T. drescheri* obtained by ethanol extraction
PEE-P	Propolis from *T. pagdeni* obtained by ethanol extraction
SI	Selective index
