# Supplementary material for: Antiviral and Anticancer Activities of Stingless Bee Propolis from Tetragonula drescheri and Tetragonula pagdeni: Toward Development of Prototype Healthcare Pharmaceuticals
Source: Int J Mol Sci. 2026 Apr 27;27(9):3855. doi: 10.3390/ijms27093855 (PMC13163722; doi:10.3390/ijms27093855)
Supplement: Supplementary file 1 [file ijms-27-03855-s001.zip › Supplementary data.pdf]

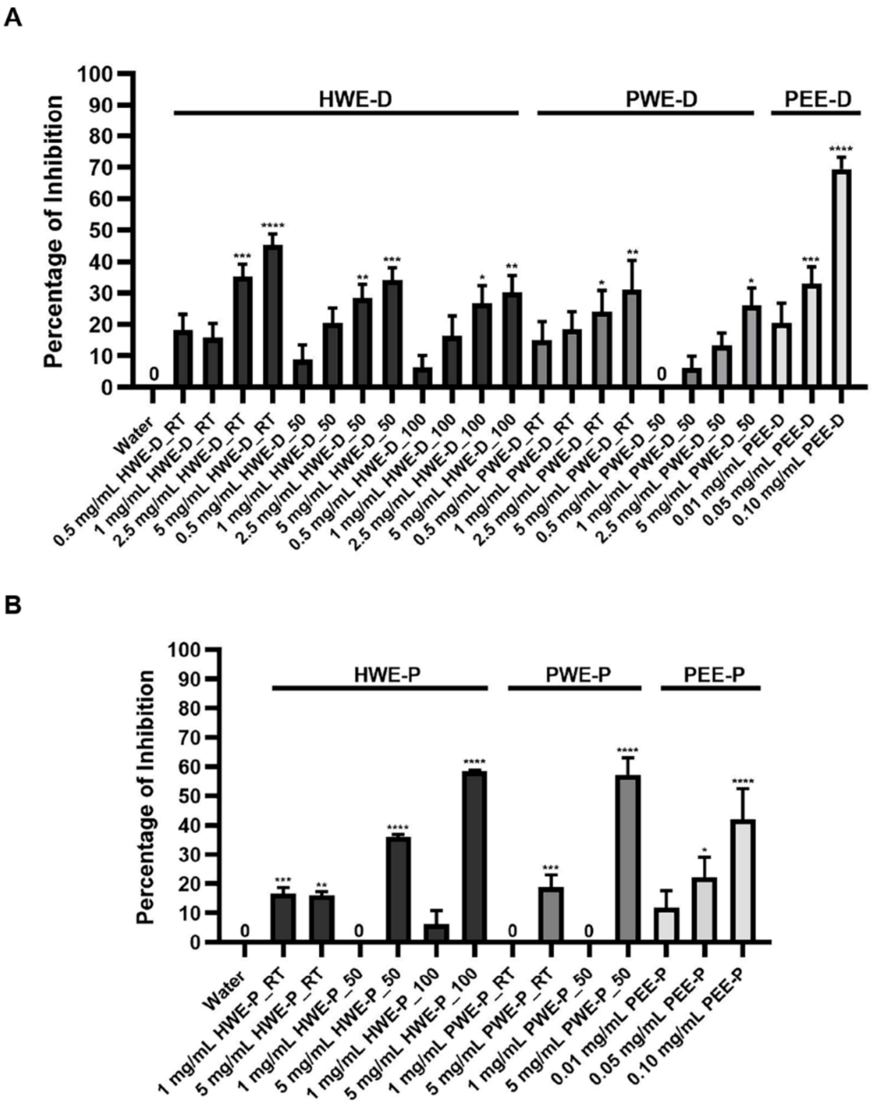


**Figure S1.** The effects of the extracts against HSV-1 KOS. (**A**) HWE-D, PWE-D, and PEE-D, and (**B**) HWE-P, PWE-P, and PEE-P were tested in HSV-1−infected Vero cells in the post-attachment step. Water was used as the control. The symbols *, **, ***, and **** indicate significant differences ($p < 0.05, 0.01, 0.001$, and $0.0001$, respectively). Bar charts represent the mean ± SEM of triplicate experiments.

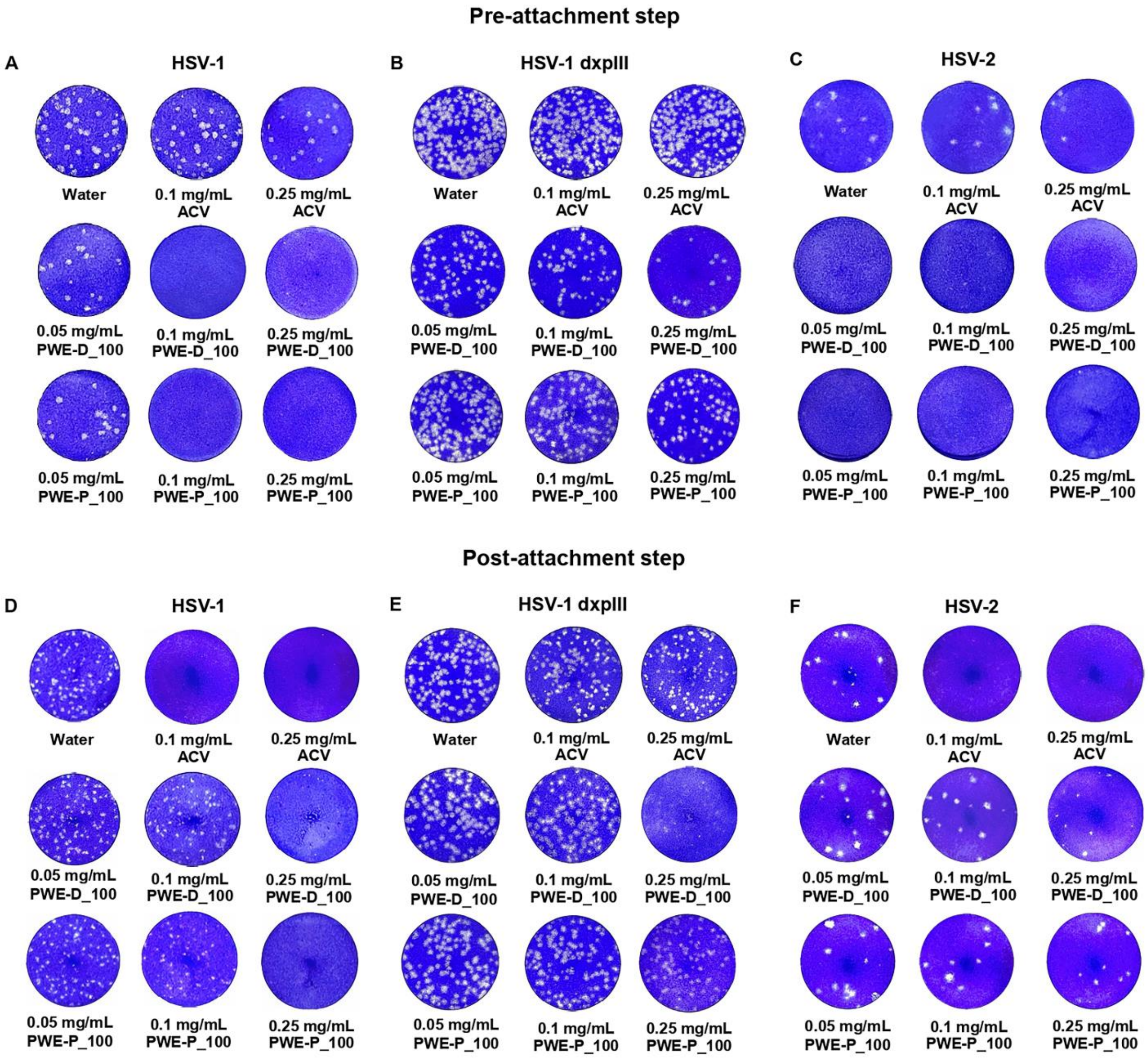


**Figure S2.** The effects of the extracts against HSV-1 KOS, HSV-1 dxpIII, and HSV-2 in the pre- and post-entry steps. The effects of PWE-D_100 and PWE-P_100 were tested in Vero cells to investigate their antiviral activities against HSV-1, HSV-1 dxpIII, and HSV-2 in (**A-C**) pre-attachment step and (**D-F**) post-attachment step. Water and ACV were used as the negative and positive controls, respectively. Plaque formation was visualized by crystal violet staining at 48-72 hours post-infection.

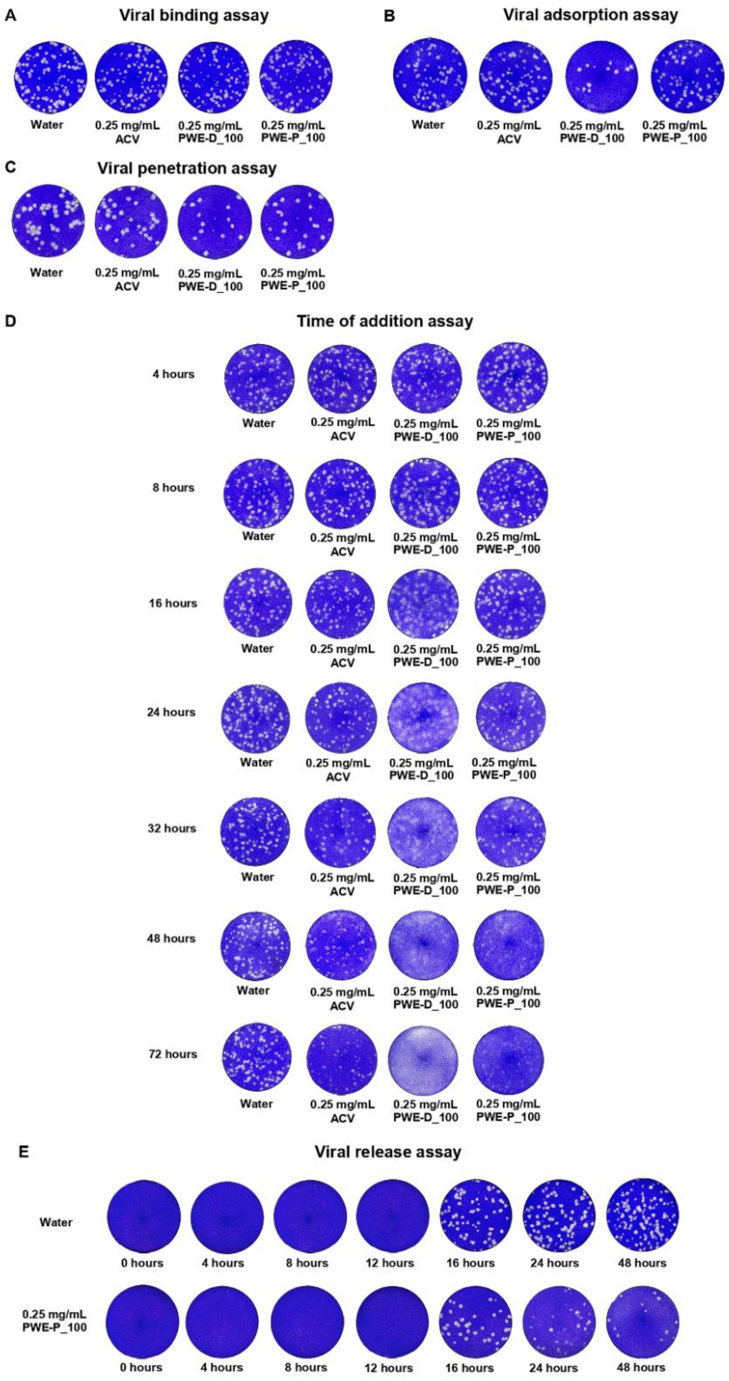


**Figure S3.** The effects of the extracts against HSV-1 dxpIII in host cell receptor binding, viral adsorption, penetration, time-of-addition of extract treatment, and viral release assays. The effects of PWE-D_100 and PWE-P_100 were tested in Vero cells to investigate their antiviral activities against HSV-1 dxpIII in (**A**) viral binding, (**B**) viral adsorption, (**C**) viral penetration, (**D**) time-of-addition (0-72 h), and (**E**) viral release assays. Water and ACV were used as the negative and positive controls, respectively. Plaque formation was stained with crystal violet at 48-72 hours post-infection.

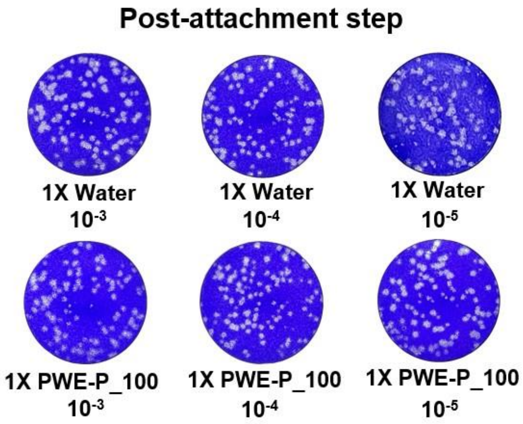


**Figure S4.** The effects of the prototype soap containing PWE-P_100 against HSV-1 dxpIII in the post-entry step. Vero cells infected with HSV-1 dxpIII were treated with 1x PWE-P_100 soaps at $10^{-3}$-$10^{-5}$ dilutions in the post-attachment. 1X Water control soaps ($10^{-3}$-$10^{-5}$) were used as controls. Plaque formation was visualized by crystal violet staining at 72 h post-infection.

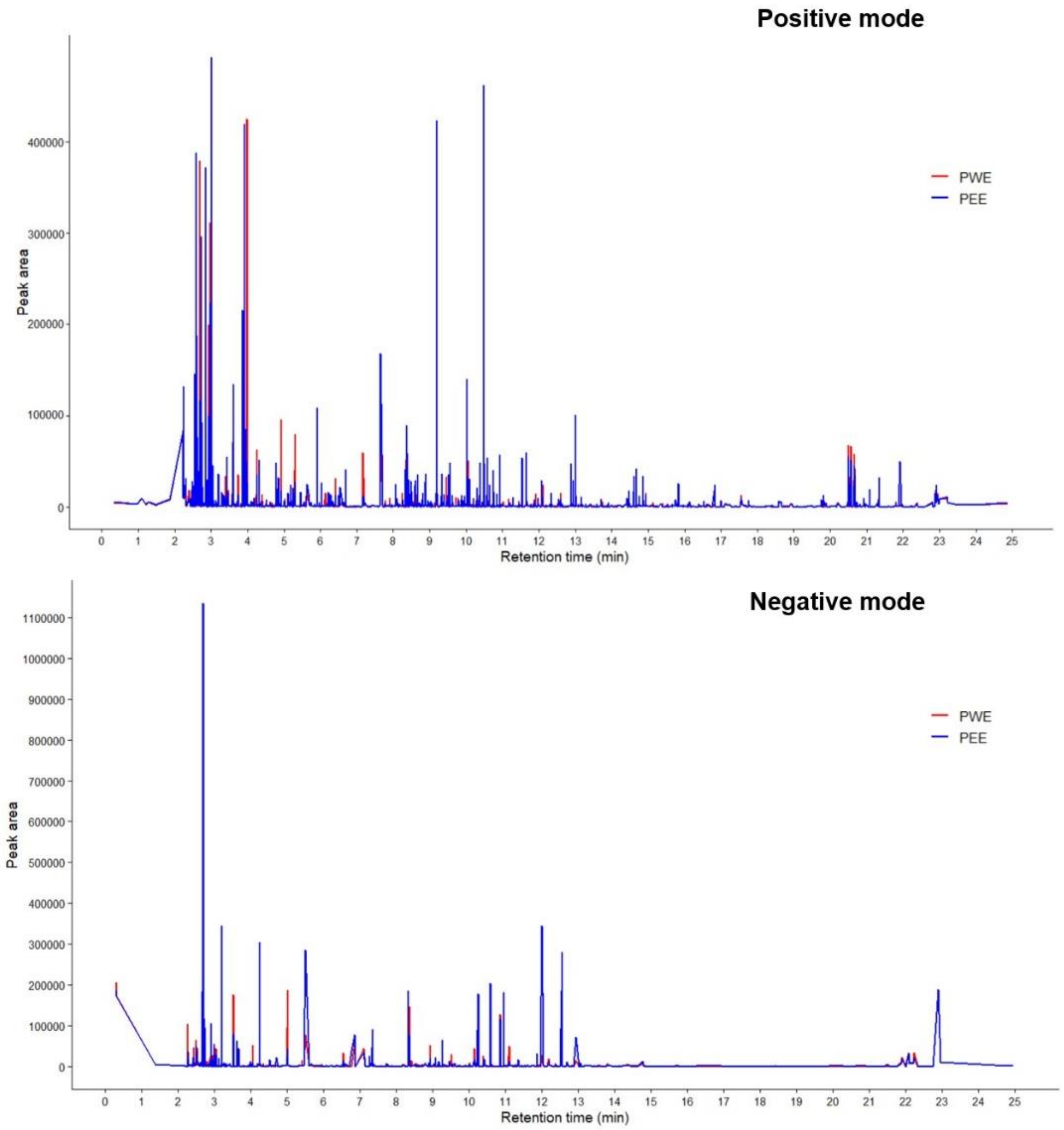


**Figure S5.** The chromatogram of PWE and PEE analyzed by ESI-Q-TOF-MS. The chromatograms of PWE and PEE were plotted between the peak area and mean retention time (min) using R version 4.5.2. Briefly, the candidate extract powder was resuspended with 0.1% formic acid prepared in LC-MS grade deionized water (CHROMASOLV®, Honeywell, Seelze, Germany). The extract solution was filtered using a 0.45 µpore size hydrophilic nylon syringe filter prior to ESI-Q-TOF-MS analysis. A volume of 100 µL of the prepared sample was transferred into an insert placed in liquid chromatography glass vials. Chemical composition analysis was performed using ESI-QTOF-MS. The separation was carried out by DIONEX Ultimate 3000 HPLC (Dionex Softron GmbH, Germering, Germany) equipped in an Acclaim Advantage II C18 (2.1 × 100 mm, 3 µm) and Acclaim Advantage II C18 (3 × 10 mm, 5 µm). Three microliters of the candidate extract were infected into the system. The column and autosampler temperature were maintained at 40

°C and 10°C, respectively. Detection was performed using a Bruker compact QTOF mass spectrometer (Bruker Daltonics, Bremen, Germany). MS signals in the m/z range of 50-1000 were acquired separately in positive and negative ion mode. The nebulizing gas pressure, drying gas flow rate, and drying gas temperature were set at 2 bars, 8 L/min, and 220°C, respectively. Chromatographic separation was conducted at flow rate of 0.3 mL/ min using a gradient program with mobile phases A (water with 0.1% formic acid) and B (acetonitrile with 0.1% formic acid). The gradient conditions were as follows: 0–2 min, 99% A and 1% B; 2–17 min, 99–1% A and 1–99% B; and 17–20 min, 1% A and 99%B. Subsequently, the gradient was changed from 99% B to 99% A between 20 and 20.1 min. From 20.1-28.5 min, the composition was maintained at 99% A and 1% B with a flow rate of 0.35 mL/min, followed by 28.5–30 min at 99% A and 1% B with a flow rate of 0.25 mL/min. Sodium formate was used as an external calibrant. The acquisition consisted of two segments: an auto MS scan from 0 to 0.3 min for sodium formate calibration and an auto MS/MS segment with fragmentation from 0.3 to 30 min. Data acquisition in both segments was performed in positive and negative ion mode at 12 Hz. The automatic in-run mass scan range was from 20 to1300 m/z. The precursor ion was set to 0.5, the number of precursors to three, the cycle time to 0.5 s, and the threshold to 400 counts. Active exclusion was applied after three spectra and released after 0.2 min. The data were processed using MetaboScape® 2022 software (Bruker, Germany). Bucketing parameters in the T-ReX 3D workflow were set as follows: an intensity threshold of 1000, a peak length of seven spectra, and peak area was used for feature quantification. Mass calibration was performed between 0–0.3 min. Feature extraction was conducted within a retention time range of 0.3 to 25 min and a mass range of 50 to 1000 m/z. Metabolite identification was performed by matching MS/MS spectra and retention times with entries in the Bruker MetaboBase Personal Library 2.0 and the MassBank of North America (MONA) database, Compounds were annotated through library matching, and metabolites with higher annotation quality (AQ) score were selected based on retention time, MS/MS score, m/z values, mSigma, and analyte spectral library matching. Quantification of the data matrix was based on the peak intensity of each metabolite. Only significant metabolites ($p < 0.05$) registered in the Bruker MetaboBase Personal Library 2.0 and MONA databases were included in the final dataset.

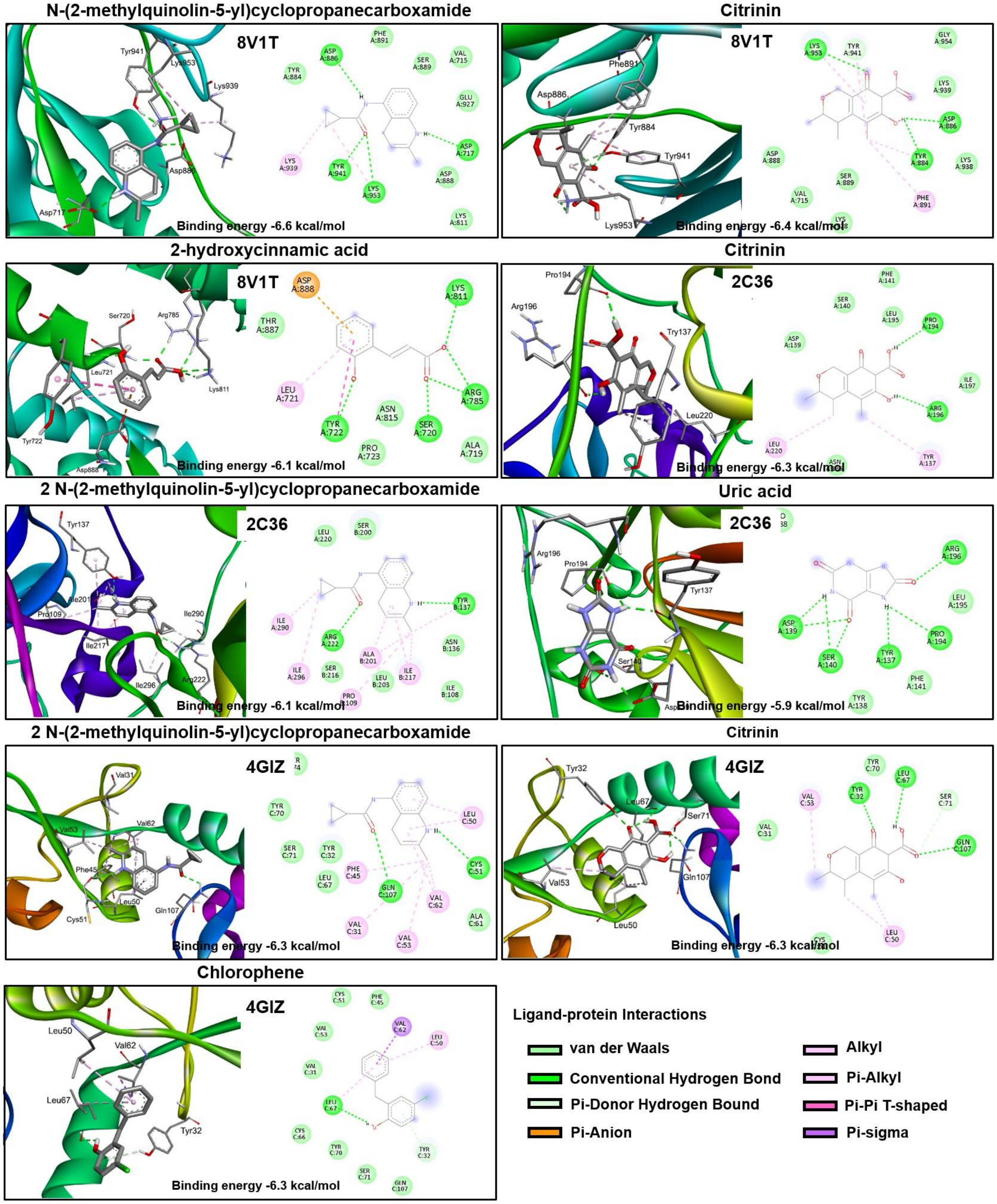


**Figure S6.** Molecular docking of N-(2-methylquinolin-5-yl)cyclopropanecarboxamide, citrinin, uric acid and 2-hydroxycinnamic acid, and chlorophene with HSV-1 DNA polymerase, HSV-1 gD, and HPV-16 E6. The receptor of viral target including HSV-1 DNA polymerase (PDB ID: 8V1T, accessed on 5 February 2025), HSV-1 gD (PDB ID: 2C36), HPV-16 E6 (PDB ID: 4GIZ, accessed on 16 March 2025), were downloaded from https://www.rcsb.org/. Acyclovir triphosphate and dextran were retrieved from 8V1T (accessed on 5 February 2025) and 5OCA (26 November 2025),

respectively. The ligands were downloaded from PubChem (https://pubchem.ncbi.nlm.nih.gov/) accessed during December 2025 to January 2026. The unnecessary molecules, including water and other non-essential atoms, were removed using BIOVIA Discovery Studio Visualizer (v21.1.0.20298; San Diego: Dassault Systèmes, 2020). Docking of receptor and ligand was performed in PyRx (Python prescription version 0.8) [1]. The receptor was processed through Make Macromolecule module, while the ligands were energy-minimized before docking to obtain stable conformations and reduce steric clashes. Then, the minimized ligands were subsequently converted into AutoDock-compatible PDBQT files using PyRx [2]. The grid box of HSV-1 DNA polymerase was X = 145.4772, Y = 145.9518, and Z= 124.1867, with dimensions in angstrom of X = 29.2038, Y = 22.0033, and Z = 29.0460; HSV-1 gD was X = 46.1348, Y = 42.8572, and Z= 97.5284, with dimensions in angstrom of X = 20.3493, Y = 20.5012, and Z = 20.9509), and HPV-16 E6 was X = -1.3241, Y = 55.6770, and Z= 25.7175, with dimensions in angstrom of X = 11.6117, Y = 13.4133, and Z = 11.3421. The most negative binding affinity (kcal/mol) with the lowest binding energy, and the minimum RMSD were collected to visualize in PyMOL (Version 2.5.7; Schrödinger LLC, New York, NY, USA) and BIOVIA Discovery Studio Visualizer.

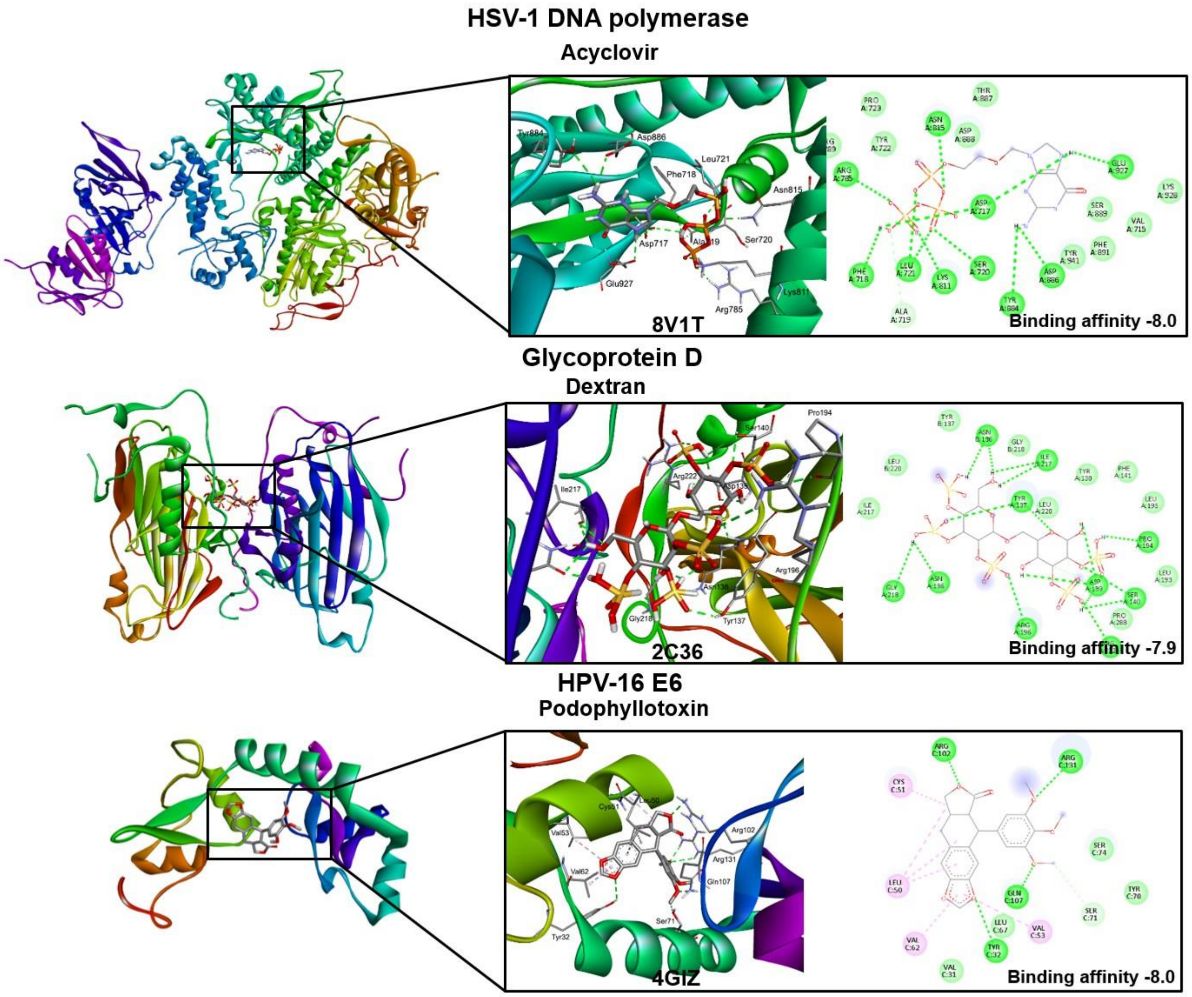


**Figure S7.** Molecular docking of positive controls, acyclovir, dextran, and podophyllotoxin, found in PWE-P_100 and PEE-D targeting HSV-1 DNA polymerase, HSV-1 gD, and HPV-16 E6.

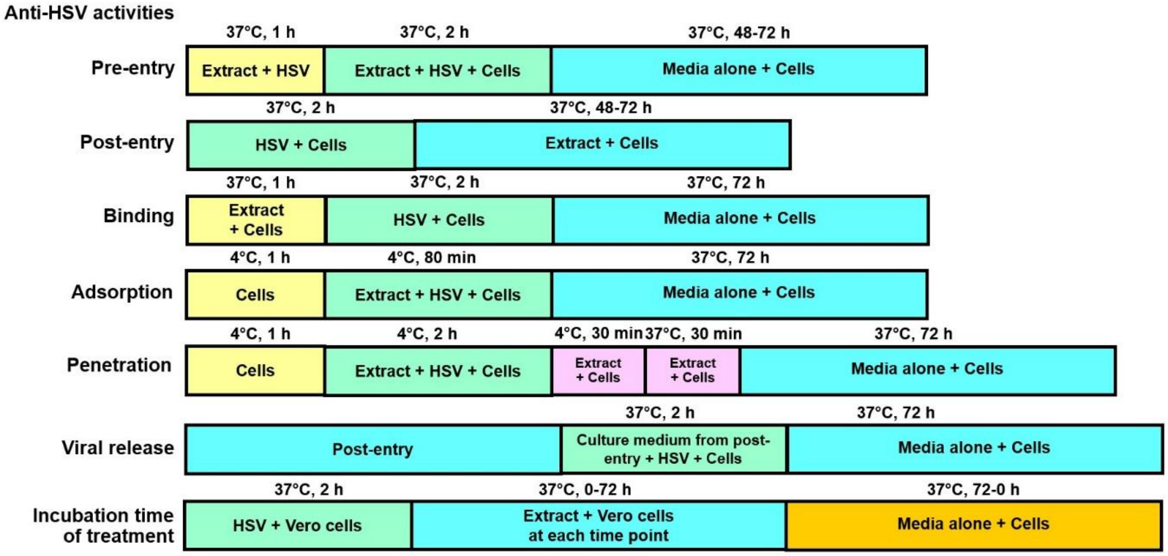


**Figure S8.** Schematic diagram of experiments involving HSV. The mechanism of action of the extracts against HSV, including the steps of pre-entry, post-entry, host cell receptor binding, viral adsorption, viral penetration, viral release and different incubation times of extract treatment were examined. For host cell receptor binding assay, Vero cells at $1\times10^5$ cells/well incubated for 24 h were treated with the extracts for 1 h, washed, and then infected with HSV (MOI 0.002) for 2 h at 37 °C. After the unbound virus was removed by PBS washing, cells were overlaid with complete medium containing 0.5% CMC and incubated for 72 h. Finally, cells were fixed with 10% formaldehyde, stained with 0.5% crystal violet, and plaques were counted to calculate the percentage of viral inhibition. For viral adsorption assay, Vero cells at $1\times10^5$ cells/well incubated for 24 h were pre-incubated at 4 °C for 1 h and then exposed to HSV (MOI 0.004) mixed with extracts at the most effective concentrations (1:1, v/v) for 80 min at 4 °C. After unbound virus was removed by PBS washing, cells were overlaid with complete medium containing 0.5% CMC and incubated for 72 h. Finally, cells were fixed with 10% formaldehyde, stained with 0.5% crystal violet, and plaques were counted to calculate the percentage of viral inhibition. For viral penetration assay, Vero cells at $1\times10^5$ cells/well incubated for 24 h were pre-incubated at 4 °C for 1 h and then infected with HSV (MOI 0.002) for 2 h at 4 °C. After being washed with cold PBS, cells were treated with extracts at the most effective concentrations for 30 min at 4 °C and immediately shifted to 37 °C for another 30 min. Unbound and adsorbed viruses were removed by sequential washes with PBS at pH 3.0 and 11.0, followed by three washes with PBS at pH 7.4. Cells were subsequently overlaid with complete medium containing 0.5% CMC and incubated for 72 h. Finally, cells were fixed with 10% formaldehyde, stained with 0.5% crystal violet, and plaques were counted to calculate the percentage of viral inhibition. For viral release assay, the culture medium from the post-entry entry was prepared in different 10-fold dilutions and incubated for 2 h. After removing residual viruses, the cells were overlaid with complete medium containing 0.5% CMC and maintained for 72 h. Finally, the cells were fixed with 10% formaldehyde, stained with 0.5% crystal violet, and plaques were counted to calculate the percentage of viral inhibition, as mentioned above. For time-of-addition assay, an HSV (MOI 0.002) was infected with Vero cells for 2 h at 37°C. Then, the extracts were subjected to cells and incubated at different time points of the extract treatment (0, 4, 8, 16, 24, 32, 48, 72 h) and subsequently replaced with medium containing 0.5% CMC for the remaining incubation period (72, 68, 64, 56, 48, 40, 24, 0 h, respectively). Finally, the cells were fixed with 10% formaldehyde, stained with 0.5% crystal violet, and plaques were counted to calculate the percentage of viral inhibition, as mentioned above.

**Table S1.** Concentration of bioactive compounds and anti-oxidation activity in stingless bee honey and propolis across 10 extracts.

| Extract | Polysaccharide (mg/mL) | Protein (mg/mL) | Tannin content (mg/mL) | DPPH activity $IC_{50}$ (mg/mL) |
|---|---|---|---|---|
| HWE-D_RT | 470.14±3.93 [a] | 1.86±0.07 [a] | 2.51±0.37 [a] | >10 |
| HWE-D_50 | 461.82±12.72 [a] | 2.06±0.10 [a] | 2.74±0.08 [a] | >10 |
| HWE-D_100 | 463.13±6.00 [a] | 2.65±1.28 [a] | 2.65±0.11 [a] | >10 |
| PWE-D_RT | 29.30±2.22 [b] | 1.09±0.02 [b] | 0.20±0.02 [b] | 6.57±0.07 [a] |
| PWE-D_50 | 30.09±1.72 [b] | 0.85±0.11 [b] | 0.16±0.08 [b] | 9.48±0.10 [b] |
| HWE-P_RT | 507.55±25.41 [a] | 1.49±0.08 [a] | 1.98±0.41 [a] | >10 |
| HWE-P_50 | 504.57±29.17 [a] | 1.15±0.07 [b] | 1.54±0.42 [c] | >10 |
| HWE-P_100 | 504.51±24.50 [a] | 1.15±0.04 [b] | 1.47±0.03 [c] | >10 |
| PWE-P_RT | 27.77±1.17 [b] | 1.25±0.03 [c] | 0.19±0.06 [b] | 6.57±0.23 [a] |
| PWE-P_50 | 22.61±0.91[c] | 1.08±0.01 [d] | 0.12±0.04 [b] | 9.60±0.02 [b] |

Note: HWE-D_RT, honey from *T. drescheri* obtained by water extraction at room temperature; HWE-D_50, honey from *T. drescheri* obtained by water extraction at 50°C; HWE-D_100, honey from *T. drescheri* obtained by water extraction at 100°C; PWE-D_RT, propolis from *T. drescheri* obtained by water extraction at room temperature; PWE-D_50, propolis from *T. drescheri* obtained by water extraction at 50°C; HWE-P_RT, honey from *T. pagdeni* obtained by water extraction at room temperature; HWE-P_50, honey from *T. pagdeni* obtained by water extraction at 50°C; HWE-P_100, honey from *T. pagdeni* obtained by water extraction at 100°C; PWE-P_RT, propolis from *T. pagdeni* obtained by water extraction at room temperature; PWE-P_50, propolis from *T. pagdeni* obtained by water extraction at 50°C. Different lowercase letters indicate significant differences among these 10 extracts.

**Table S2.** $CC_{50}$ values (mean ± SEM) of honey (HWE) and propolis (PWE) extracts from *T. drescheri* and *T. pagdeni* in Vero, 293FT, CaSki, and HeLa cells.

| Extract | Incubation time (Hours) | 50 % Cytotoxic concentration ($CC_{50}$), mg/mL | | | |
|---|---|---|---|---|---|
| | | **Vero** | **293FT** | **CaSki** | **HeLa** |
| HWE-D_RT | 48 | >25 | N/A | N/A | N/A |
| HWE-D_50 | 48 | >25 | N/A | N/A | N/A |
| HWE-D_100 | 48 | >25 | N/A | N/A | N/A |
| PWE-D_RT | 48 | >10 | N/A | N/A | N/A |
| PWE-D_50 | 48 | >10 | N/A | N/A | N/A |
| HWE-P_RT | 48 | >25 | N/A | N/A | N/A |
| HWE-P_50 | 48 | >25 | N/A | N/A | N/A |
| HWE-P_100 | 48 | >25 | N/A | N/A | N/A |
| PWE-P_RT | 48 | >10 | N/A | N/A | N/A |
| PWE-P_50 | 48 | >10 | N/A | N/A | N/A |

* N/A denotes "Not available".

**Table S3.** Formulation of prototype herbal soap.

| **Ingredients** | **Formulation (20 mL)** | | **Uses** |
|---|---|---|---|
| | **1X** | **2X** | |
| Ammonium Lauryl Sulfate | 2.0 g | 4.0 | Surfactant |
| Carboxymethyl Cellulose | 0.8 g | 1.6 | Thickening agent |
| Comperlan KD | 0.2 g | 0.4 | Surfactant |
| Glycerin | 0.2 g | 0.4 | Humectant |
| Citric acid | 0.01 g | 0.02 | pH Adjuster |
| Distilled water | Adjust to 20 mL | Adjust to 20 mL | Vehicle |
| Formula | 1X | 2X | Uses |
| 1. DMSO-control soap | | | Control for analyzing HPV-16/18 *E6* mRNA |
| Soap | 0.9 mL | 0.5 mL | |
| DMSO | 0.1 mL | 0.5 mL | |
| 2. PEE-D soap | | | Test group for analyzing HPV-16/18 *E6* mRNA |
| Soap | 0.9 mL | 0.5 mL | |
| 500 mg/mL of extract | 0.1 mL | 0.5 mL | |
| 3. Water-control soap | | | Control for analyzing plaque assay of HSV-1 dxpIII |
| Soap | 0.9 mL | 0.5 mL | |
| 500 mg/mL of extract | 0.1 mL | 0.5 mL | |
| 4. PWE-P_100 soap | | | Test group for analyzing plaque assay of HSV-1 dxpIII |
| Soap | 0.9 mL | 0.5 mL | |
| 500 mg/mL of extract | 0.1 mL | 0.5 mL | |

**Table S4.** Prediction of drug-likeness of candidate chemical compounds found in PWE-D_100 and PEE-D.

| Compound | CID | Formula | RB | HBA | HBD | TPS Å² | Log Po/w | Water Solubility | Drug likeness |
|---|---|---|---|---|---|---|---|---|---|
| | | | | PEE-D in positive mode | | | | | |
| Proline betaine | 7016562 | $C_7H_{13}NO_2$ | 1 | 2 | 0 | 40.13 | -1.71 | Soluble | Yes |
| N-(2-methylquinolin-5-yl)cyclopropanecarboxamide | 652673 | $C_{14}H_{14}N_2O$ | 3 | 2 | 1 | 41.99 | 2.32 | Moderately soluble | Yes |
| Phenylalanine | 6140 | $C_9H_{11}NO_2$ | 3 | 3 | 2 | 63.32 | -0.01 | Soluble | Yes |
| Adenine | 190 | $C_5H_5N_5$ | 0 | 3 | 2 | 80.48 | -0.2 | Soluble | Yes |
| Choline | 305 | $C_5H_{13}NO$ | 2 | 1 | 1 | 20.23 | -1.86 | Soluble | Yes |
| L-Proline | 145742 | $C_5H_9NO_2$ | 1 | 3 | 2 | 49.33 | -0.92 | Soluble | Yes |
| Indoline | 10328 | $C_8H_9N$ | 0 | 0 | 1 | 12.03 | 1.73 | Soluble | Yes |
| Betonicine | 164642 | $C_7H_{13}NO_3$ | 1 | 3 | 1 | 60.36 | -2.46 | Soluble | Yes |
| Indole-3-ethanol | 10685 | $C_{10}H_{11}NO$ | 2 | 1 | 2 | 36.02 | 1.64 | Soluble | Yes |
| Pipecolic acid | 849 | $C_6H_{11}NO_2$ | 1 | 3 | 2 | 49.33 | -0.61 | Soluble | Yes |
| D-Sorbitol | 5780 | $C_6H_{14}O_6$ | 5 | 6 | 6 | 121.38 | -1.9 | Soluble | Yes |
| 2-Pyrrolidinone | 12025 | $C_4H_7NO$ | 0 | 1 | 1 | 29.10 | 0.1 | Soluble | Yes |
| Arginine | 6322 | $C_6H_{14}N_4O_2$ | 5 | 4 | 4 | 127.72 | -2.04 | Soluble | Yes |
| Trigonelline | 5570 | $C_7H_7NO_2$ | 1 | 2 | 0 | 44.01 | -0.61 | Soluble | Yes |
| 1-(1,3-benzodioxol-5-ylcarbonyl)piperidine | 1370 | $C_{13}H_{15}NO_3$ | 2 | 3 | 0 | 38.77 | 2.27 | Soluble | Yes |
| L-Valine | 6287 | $C_5H_{11}NO_2$ | 2 | 3 | 2 | 63.32 | -0.78 | Soluble | Yes |
| Dimethyl fumarate | 637568 | $C_6H_8O_4$ | 4 | 4 | 0 | 52.60 | 0.48 | Soluble | Yes |
| 2-hydroxycinnamic acid | 637540 | $C_9H_8O_3$ | 2 | 3 | 2 | 57.53 | 1.4 | Soluble | Yes |
| Betaine | 115244 | $C_5H_{11}NO_2$ | 2 | 2 | 0 | 40.13 | -2.18 | Soluble | Yes |

| Compound | CID | Formula | RB | HBA | HBD | TPS Å² | Log Po/w | Water Solubility | Drug likeness |
|---|---|---|---|---|---|---|---|---|---|
| L-Glutamine | 5961 | $C_5H_{10}N_2O_3$ | 4 | 4 | 3 | 106.41 | -1.82 | Soluble | Yes |
| PWE-P_100 in positive mode | | | | | | | | | |
| Guanine | 135398634 | $C_5H_5N_5O$ | 0 | 3 | 3 | 100.45 | -0.61 | Soluble | Yes |
| N-(2-methylquinolin-5-yl)cyclopropanecarboxamide | 652673 | $C_{14}H_{14}N_2O$ | 3 | 2 | 1 | 41.99 | 2.32 | Moderately soluble | Yes |
| Betaine | 115244 | $C_5H_{11}NO_2$ | 2 | 2 | 0 | 40.13 | -2.18 | Soluble | Yes |
| Indole-3-ethanol | 10685 | $C_{10}H_{11}NO$ | 2 | 1 | 2 | 36.02 | 1.64 | Soluble | Yes |
| Phenylalanine | 6140 | $C_9H_{11}NO_2$ | 3 | 3 | 2 | 63.32 | -0.01 | Soluble | Yes |
| Choline | 305 | $C_5H_{13}NO$ | 2 | 1 | 1 | 20.23 | -1.86 | Soluble | Yes |
| Indoline | 10328 | $C_8H_9N$ | 0 | 0 | 1 | 12.03 | 1.73 | Soluble | Yes |
| L-Proline | 145742 | $C_5H_9NO_2$ | 1 | 3 | 2 | 49.33 | -0.92 | Soluble | Yes |
| Gentiannine | 354616 | $C_{10}H_9NO_2$ | 1 | 3 | 0 | 39.19 | 1.67 | Soluble | Yes |
| Adenine | 190 | $C_5H_5N_5$ | 0 | 3 | 2 | 80.48 | -0.2 | Soluble | Yes |
| Pipecolic acid | 849 | $C_6H_{11}NO_2$ | 1 | 3 | 2 | 49.33 | -0.61 | Soluble | Yes |
| D-Sorbitol | 5780 | $C_6H_{14}O_6$ | 5 | 6 | 6 | 121.38 | -1.9 | Soluble | Yes |
| Glycerophosphocholine | 11234 | $C_8H_{20}NO_6P$ | 8 | 6 | 2 | 108.86 | -2.86 | Soluble | Yes |
| Dimethyl fumarate | 637568 | $C_6H_8O_4$ | 4 | 4 | 0 | 52.60 | 0.48 | Soluble | Yes |
| Betonicine | 164642 | $C_7H_{13}NO_3$ | 1 | 3 | 1 | 60.36 | -2.46 | Soluble | Yes |
| Uric acid | 1175 | $C_5H_4N_4O_3$ | 0 | 3 | 4 | 114.37 | -0.72 | Soluble | Yes |
| 2-hydroxycinnamic acid | 637540 | $C_9H_8O_3$ | 2 | 3 | 2 | 57.53 | 1.4 | Soluble | Yes |
| Umbelliferone | 5281426 | $C_9H_6O_3$ | 0 | 3 | 1 | 50.44 | 1.51 | Soluble | Yes |
| 2-Pyrrolidinone | 12025 | $C_4H_7NO$ | 0 | 1 | 1 | 29.10 | 0.1 | Soluble | Yes |
| Hypoxanthine | 135398638 | $C_5H_4N_4O$ | 0 | 3 | 2 | 74.43 | -0.17 | Soluble | Yes |

| Compound | CID | Formula | RB | HBA | HBD | TPS Å² | Log Po/w | Water Solubility | Drug likeness |
|---|---|---|---|---|---|---|---|---|---|
| | | | | PEE-D in negative mode | | | | | |
| Gluconic acid | 10690 | $C_6H_{12}O_7$ | 5 | 7 | 6 | 138.45 | -2.42 | Soluble | Yes |
| 2-Hydroxycaproic acid | 99824 | $C_6H_{12}O_3$ | 4 | 3 | 2 | 57.53 | 0.61 | Soluble | Yes |
| Citric acid | 311 | $C_6H_8O_7$ | 5 | 7 | 4 | 132.13 | -1.51 | Soluble | Yes |
| L-Lactic acid | 107689 | $C_3H_6O_3$ | 1 | 3 | 2 | 57.53 | -0.59 | Soluble | Yes |
| Pipecolic acid | 849 | $C_6H_{11}NO_2$ | 1 | 3 | 2 | 49.33 | -0.61 | Soluble | Yes |
| 3-Phenyllactate | 4060207 | $C_9H_{10}O_3$ | 3 | 3 | 2 | 57.53 | 1.02 | Soluble | Yes |
| 2-methyl-3-Pyrimidin-2-yl-Propionic Acid | 42614418 | $C_8H_{10}N_2O_2$ | 3 | 4 | 1 | 63.08 | 0.69 | Soluble | Yes |
| Hydroxyphenyllactic acid | 9877544 | $C_9H_{10}O_4$ | 2 | 4 | 3 | 77.76 | 0.46 | Soluble | Yes |
| benzoin | 8400 | $C_{14}H_{12}O_2$ | 3 | 2 | 1 | 37.30 | 2.32 | Moderately soluble | Yes |
| L-Valine | 6287 | $C_5H_{11}NO_2$ | 2 | 3 | 2 | 63.32 | -0.78 | Soluble | Yes |
| Citrinin | 54680783 | $C_{13}H_{14}O_5$ | 1 | 5 | 2 | 83.83 | 1.3 | Soluble | Yes |
| Etidronate | 3305 | $C_2H_8O_7P_2$ | 2 | 7 | 5 | 154.91 | -2.36 | Soluble | No |
| 2-BENZYL-4-CHLOROPHENOL 95% (Chlorophene) | 8425 | $C_{13}H_{11}ClO$ | 2 | 1 | 1 | 20.23 | 3.57 | Moderately soluble | Yes |
| 2-Acetamido-2-deoxy-beta-D-glucosylamine | 46779877 | $C_8H_{16}N_2O_5$ | 3 | 6 | 5 | 125.04 | -2.07 | Soluble | Yes |
| SNAP | 6603945 | $C_7H_{12}N_2O_4S$ | 6 | 5 | 2 | 121.13 | 0.23 | Soluble | Yes |
| Trp-Asn | 18218248 | $C_{15}H_{18}N_4O_4$ | 8 | 5 | 5 | 151.30 | -0.78 | Soluble | No |
| Mucate | ChemSpider ID | $C_6H_{10}O_8$ | 5 | 8 | 6 | 155.52 | -2.43 | Soluble | No |

| Compound | CID | Formula | RB | HBA | HBD | TPS Å² | Log Po/w | Water Solubility | Drug likeness |
|---|---|---|---|---|---|---|---|---|---|
| | 2301286 | | | | | | | | |
| 2-Isopropylmalate | 58 | $C_7H_{12}O_5$ | 4 | 5 | 3 | 94.83 | -0.08 | Soluble | Yes |
| Pyrogallol | 1057 | $C_6H_6O_3$ | 0 | 3 | 3 | 60.69 | 0.58 | Soluble | Yes |
| Biotin | 171548 | $C_{10}H_{16}N_2O_3S$ | 5 | 3 | 3 | 103.73 | 0.59 | Soluble | Yes |
| PWE-P_100 in negative mode | | | | | | | | | |
| Gluconic acid | 10690 | $C_6H_{12}O_7$ | 5 | 7 | 6 | 138.45 | -2.42 | Soluble | Yes |
| Citrinin | 54680783 | $C_{13}H_{14}O_5$ | 1 | 5 | 2 | 83.83 | 1.3 | Soluble | Yes |
| Hydroxyphenyllactic acid | 9877544 | $C_9H_{10}O_4$ | 2 | 4 | 3 | 77.76 | 0.46 | Soluble | Yes |
| L-Valine | 6287 | $C_5H_{11}NO_2$ | 2 | 3 | 2 | 63.32 | -0.78 | Soluble | Yes |
| Uric acid; LC-tDDA; CE40 | 1175 | $C_5H_4N_4O_3$ | 0 | 3 | 4 | 114.37 | -0.72 | Soluble | Yes |
| Malic acid | 525 | $C_4H_6O_5$ | 3 | 5 | 3 | 94.83 | -1 | Soluble | Yes |
| Trp-Asn | 18218248 | $C_{15}H_{18}N_4O_4$ | 8 | 5 | 5 | 151.30 | -0.78 | Soluble | No |
| 3-Hydroxy-3-methylglutarate | 1162 | $C_6H_{10}O_5$ | 4 | 5 | 3 | 94.83 | -0.49 | Soluble | Yes |
| 2-methyl-3-Pyrimidin-2-yl-Propionic Acid | 42614418 | $C_8H_{10}N_2O_2$ | 3 | 4 | 1 | 63.08 | 0.69 | Soluble | Yes |
| 2-Isopropylmalate | 77 | $C_7H_{12}O_5$ | 4 | 5 | 3 | 94.83 | -0.08 | Soluble | Yes |
| 2-BENZYL-4-CHLOROPHENOL 95% (Chlorophene) | 8425 | $C_{13}H_{11}ClO$ | 2 | 1 | 1 | 20.23 | 3.57 | Moderately soluble | Yes |
| Citric acid | 311 | $C_6H_8O_7$ | 5 | 7 | 4 | 132.13 | -1.51 | Soluble | Yes |
| Lys-Lys | 128837 | $C_{12}H_{26}N_4O_3$ | 12 | 6 | 5 | 144.46 | -0.99 | Soluble | No |
| Threonic acid | 5460407 | $C_4H_8O_5$ | 3 | 5 | 4 | 97.99 | -1.58 | Highly soluble | Yes |

| Compound | CID | Formula | RB | HBA | HBD | TPS Å$^2$ | Log Po/w | Water Solubility | Drug likeness |
|---|---|---|---|---|---|---|---|---|---|
| Pipecolic acid | 849 | $C_6H_{11}NO_2$ | 1 | 3 | 2 | 49.33 | -0.61 | Soluble | Yes |
| Glucose | 5793 | $C_6H_{12}O_6$ | 1 | 6 | 5 | 110.38 | -2.23 | Soluble | Yes |
| Ortophosphate | 71181 | $H_3O_4P$ | 0 | - | - | 87.57 | - | - | - |
| Etidronate | 3305 | $C_2H_8O_7P_2$ | 2 | 7 | 5 | 154.91 | -2.36 | Soluble | No |
| Methylmalonate | 487 | $C_4H_6O_4$ | 2 | 4 | 2 | 74.60 | -0.23 | Soluble | Yes |
| Monomethyl phthalate | 20392 | $C_9H_8O_4$ | 3 | 4 | 1 | 63.60 | 1.27 | Soluble | Yes |

**Table S5.** Primer sequence.

| Primer | Primer sequence |
|---|---|
| BAX | Forward: 5′-CCCGAGAGGTCTTTTTCCGAG-3′<br>Reverse: 5′-CCAGCCCATGATGGTTCTGAT-3′ |
| GAPDH | Forward: 5′-TCATCAGCAATGCCTCCTGCA-3′<br>Reverse: 5′-TGGGTAGCAGTGATGGCA-3′ |
| HPV16E6 | Forward: 5′-TCAAAAGCCACTGTGTCCTG-3′<br>Reverse: 5′-CAGCTGGGTTTCTCTACGTGT-3′ |
| HPV18E6 | Forward: 5′-GTAACCGAAAACGGTCGGGA-3′<br>Reverse: 5′-AGTTCCGTGCACAGATCAGG-3′ |
| HSV-gD | Forward: 5′-AGCAGGGGTTAGGGAGTTGT-3′<br>Reverse: 5′-CCATCTTGAGAGAGGCATCC-3′ |
| HSV-ICP4 | Forward: 5′-CGACACGGATCCACGACCC-3′<br>Reverse: 5′-GATCCCCCTCCCGCGCTTCGTCCG-3′ |
| HSV-UL30 | Forward: 5′-GTGTTGTGCCGCGGTCTCAC-3′<br>Reverse: 5′-GGTGAACGTCTTTTCGAACTC-3′ |

**Reference**


1. Dallakyan, S.; Olson, A. J., Small-molecule library screening by docking with PyRx. In *Chemical biology: methods and protocols*, Springer: 2014; pp 243-250.
2. Trott, O.; Olson, A. J., AutoDock Vina: improving the speed and accuracy of docking with a new scoring function, efficient optimization, and multithreading. *Journal of Computational Chemistry* **2010,** *31*, 455-461.
